# Gene expression profiling enables refined parcellation of cortical layers in the heterogeneous human cerebral cortex

**DOI:** 10.1186/s13073-026-01704-z

**Published:** 2026-07-08

**Authors:** Yanrong Wei, Youzhe He, Yuyang Liu, Langjian Zhu, Tiannan Feng, Zhiming Shen, Wu Wei, Longqi Liu, Lei Han, Lifang Wang

**Affiliations:** 1 College of Life Sciences, University of Chinese Academy of Sciences, Beijing, 100049 China; 2 State Key Laboratory of Genome and Multi-omics Technologies, BGI Research, Hangzhou, 310030 China; 3 Institute of Neuroscience, State Key Laboratory of Brain Cognition and Brain-inspired Intelligence Technology, CAS Center for Excellence in Brain Science and Intelligence Technology, University of Chinese Academy of Sciences, Chinese Academy of Sciences, Shanghai, 200031 China; 4 Shanghai Center for Brain Science and Brain-Inspired Technology, Shanghai, 201602 China; 5 Lingang Laboratory, Shanghai, China; 6 CAS Key Laboratory of Computational Biology, Shanghai Institute of Nutrition and Health, University of Chinese Academy of Sciences, Chinese Academy of Sciences, Shanghai, 200031 China; 7 Key Laboratory of Brain Cell Mapping of Zhejiang Province, BGI Research, Hangzhou, 310030 China; 8 Key Laboratory of Spatial Omics of Zhejiang Province, BGI Research, Hangzhou, 310030 China

**Keywords:** Human cortex, Gene expression-defined cortical layers, Stereo-seq, Layer parcellation, Cross-species, Laminar disorganization

## Abstract

**Background:**

Precise delineation of cortical layers is fundamental for understanding human brain organization, cell-type architecture, and disease-related tissue alterations. However, traditional anatomy-based methods often lack molecular resolution and suffer from inter-observer subjectivity.

**Methods:**

Here, we present gene expression-defined cortical layers (GD-Ls) using the BayesSpace algorithm, a high-resolution framework for cortical parcellation based on spatial transcriptomics.

**Results:**

Compared with traditional anatomy-based approaches, GD-Ls more accurately resolve laminar boundaries and capture fine-scale laminar heterogeneity, including sublayer-like domains within L1, L3, and L6, as well as a molecularly distinct transition zone at the gray-white matter interface. Validation across diverse cortical lobes, multiple spatial platforms, and independent healthy postmortem datasets demonstrates that GD-Ls capture the intrinsic molecular architecture of the cortex irrespective of tissue source. Furthermore, cross-species analyses show that this framework is extensible to macaque and mouse cortices. Crucially, GD-Ls successfully identify subtle laminar disorganization and aberrant cellular and molecular signatures in pathologically altered tissues, which are often missed by conventional histology.

**Conclusions:**

Together, GD-Ls provide an objective and reproducible tool for standardized cortical mapping and for identifying early pathological signatures in the human brain. The source code is available on GitHub (https://github.com/YanrongWei/GD-Ls).

**Supplementary Information:**

The online version contains supplementary material available at 10.1186/s13073-026-01704-z.

## Background

The cerebral cortex exhibits a highly conserved six-layer laminar organization that underlies essential cognitive functions across mammals [1, 2]. Although classical neuroanatomical studies have described cortical cytoarchitecture in detail, delineation of interlaminar boundaries has largely relied on manual annotation, which is inherently subject to observer-dependent variability. As a result, achieving precise and reproducible cortical parcellation remains a major challenge.

To investigate the cellular composition and functional organization of cortical layers, a variety of approaches have been used in single-nucleus RNA sequencing (snRNA-seq) and spatial transcriptomics (ST) studies. For example, Hodge et al. (2019) performed transcriptomic analyses on microdissected human cortical layers guided by Nissl staining using Smart-seq v4 [3], enabling the identification of layer-specific cell types and providing a foundational view of the cellular architecture of the human cortex. Subsequent studies have adopted anatomically guided strategies to further characterize molecular and cellular features across human and other mammalian cortices [4–7]. However, dissection-dependent techniques remain vulnerable to boundary misidentification due to technical artifacts inherent to manual microdissection.

Several studies have also attempted to delineate cortical laminar boundaries using layer-enriched gene expression patterns [8, 9]. Notably, Huuki-Myers et al. (2024) showed that data-driven approaches can improve cortical layer delineation relative to traditional methods (9). Their analyses also identified the vascular-rich meninges, which can be difficult to distinguish from layer 1. However, they did not directly compare molecularly defined boundaries with anatomically defined layers, an important step toward bridging classical neuroanatomy and molecular neuroscience.

In this study, we applied the GD-Ls parcellation approach to evaluate laminar boundaries in comparison with anatomically defined layers using 15 Stereo-seq sections from four cortical lobes of 14 surgical donors. Relative to conventional anatomy-based methods, GD-Ls showed improved adaptability to both linear and curved laminar architectures and facilitated the identification of pathologically altered regions, including tumor-infiltrated areas. Spatial cell-type distributions further supported the utility of GD-Ls for layer delineation. Together, these results suggest that gene expression-based parcellation provides a sensitive and broadly applicable framework for refining cortical lamination and improving the detection of cortical pathology.

## Methods

### Human cortical tissue cohort and sample collection

The dataset comprising all samples analyzed in this study was obtained from the companion study [10]. Briefly, the collection and use of all samples were approved by the Institutional Review Board of the BGI Group (No. BGI-IRB 24104). Relatively unaffected human cortical tissues, excluding tumor foci and obvious lesion areas, were obtained from surgical patients with clinical/pathological diagnoses of tumor, epilepsy, or abscess (Supplementary Material 1: Table S1). In total, 15 cortical sections from 14 donors were included, spanning four cortical lobes: frontal lobe (1 section), parietal lobe (6 sections), temporal lobe (6 sections), and occipital lobe (2 sections) (Supplementary Material 2: Fig. S1A). The two occipital lobe sections were adjacent slices from the same donor and were processed as technical replicates to assess reproducibility. All cortical sections were subjected to spatial transcriptomic profiling. Downstream analyses performed on these sections included gene expression profiling, GD-Ls-based cortical layer parcellation, identification of layer-associated molecular features, and cross-section comparisons of laminar organization.

### Anatomy-based parcellation

To guide the manual parcellation of cortical layers, immunohistochemical staining for the neuron-specific nuclear protein (NeuN) was performed on adjacent frozen sections according to a standardized protocol. Briefly, sections were thawed, fixed in 4% PFA, and permeabilized with 0.5% Triton X-100. After blocking with 10% serum, sections were incubated overnight at 4 °C with a primary antibody against NeuN (1:1500 dilution). The following day, endogenous peroxidase activity was quenched with 0.6% H₂O₂. After washing, antigen-antibody complexes were detected using a species-appropriate secondary antibody, followed by incubation with an ABC kit and development with a DAB substrate. Finally, the stained sections were dehydrated through an ethanol series, cleared in toluene, and mounted with DPX.

Based on the resulting NeuN staining patterns and the characteristic differences in neuronal density, the cortical layers (L1-L6) and white matter were manually delineated to establish an anatomically grounded layered structure.

### Datasets preprocessing

Data quality was strictly controlled; all 15 sections were required to exhibit at least 1,000 detected genes per 50 × 50 µm^2^ area (Bin100 resolution) to ensure sufficient transcriptomic depth for laminar parcellation (Supplementary Material 2: Fig. S1B; Supplementary Material 1: Table S2). The expression profile matrix was then binned into Bin50, Bin100, Bin150, Bin200 and Bin300, consolidating the transcripts of the same gene within each bin. In the subsequent analysis, each bin size was treated as a spatial spot (approximating a pseudocell).

Meanwhile, we used ssDNA images from the same section to segment cells and obtained cell bin data. However, the section II-5 failed to obtain segmented cell bin data due to the poor quality of the ssDNA staining image.

The snRNA-seq data from human cortex were applied an iterative clustering strategy adapted from the scrattch.hicat package (https://github.com/AllenInstitute/scrattch.hicat), in which batch effects across libraries were corrected at each iteration using the Harmony algorithm. Final cell type annotations were based on subclass-specific marker genes.

### Sensitivity analysis for bin-size selection

To determine an appropriate bin size for cortical laminar analysis, we systematically compared clustering results generated from multiple bin sizes (Bin50, Bin100, Bin150, Bin200, and Bin300). For each bin size, BayesSpace clustering was performed using the same clustering parameter, and the resulting clusters were evaluated using four complementary metrics. Firstly, concordance between BayesSpace clusters and manually annotated cortical layers was assessed using the adjusted Rand index (ARI). ARI quantifies the similarity between two label assignments while correcting for chance agreement. Secondly, we calculated the normalized mutual information (NMI) between BayesSpace clusters and manually annotated layers. NMI measures the amount of shared information between two clustering solutions and was used as an additional indicator of laminar concordance. Thirdly, we evaluated cluster purity with respect to manual layers. Purity was calculated as the fraction of observations in each BayesSpace cluster belonging to the dominant manually annotated layer, and then summarized across all clusters for each bin size. Higher purity indicates that clusters are more specifically associated with individual cortical layers. Finally, we quantified spatial coherence to assess the local spatial continuity of clustering results. For each section, the proportion of immediately adjacent bins (four-neighborhood) assigned to the same BayesSpace cluster was calculated, and the mean value was used as the spatial coherence score. Higher values indicate greater local continuity of cluster assignments. For analyses involving multiple tissue sections, these metrics were first calculated separately for each section and then summarized across sections as mean values. Together, these four metrics were used to assess the balance between laminar concordance, cluster specificity, and spatial continuity across different bin sizes.

### Unsupervised clustering and benchmarking for cortical parcellation

To identify a robust unsupervised clustering approach for delineating cortical layers in human cortex Stereo-seq data, we systematically evaluated seven algorithms: BayesSpace (v1.6.0) with Harmony (v0.1.0) batch correction, SpaGCN (v1.2.0), spatially-constrained clustering (SCC), graph-based clustering, Louvain clustering, GraphST-based spatial clustering and BANKSY [11–17]. We quantitatively assessed clustering accuracy against anatomy-defined layers parcellation (AD-Ls) through the ARI, where higher ARI values indicate greater similarity between gene-expression defined cortical layers (GD-Ls) and AD-Ls. Among the tested algorithms, BayesSpace with Harmony batch correction demonstrated superior performance in GD-Ls that aligned with AD-Ls.

The detailed implementation of each algorithm is as follows: (1) BayesSpace: To enable multi-section joint analysis, we performed planar alignment of tissue sections using the BayesSpace joint clustering workflow. Spatial domain identification was executed via the *spatialCluster()* function with 10, 000 iterations (*nrep* = 10,000). The number of clusters (q) was tested ranging from 2 to 24. Bayesian spatial clustering was performed using the first 30 Harmony-corrected principal components (PCs, Harmony v0.1.1) as input. (2) SpaGCN: We followed the standard pipeline to assign spatial spots into seven GD-Ls per section. The model was constructed through sequential executions of *SpaGCN()*, *set_l()*, and *train()*- until seven stable domains were resolved. Default parameters were maintained unless otherwise specified. (3) SCC: SCC was implemented using a modified *Leiden* algorithm. The input K-nearest neighbor (KNN) network was fused with a spatial neighbor network, where the *s_neigh* hyperparameter (set to 8) modulated the weight of spatial proximity in cluster assignments. (4) Graph-based clustering: Joint analysis across all sections were performed using *buildSNNGraph()* in the scran package, followed by the Walktrap community detection algorithm via igraph (v1.2.4.1). A shared nearest-neighbor (SNN) graph was constructed based on 10 nearest neighbors using the first 30 harmony-corrected dimensions and partitioned into 7 clusters. (5) Louvain clustering: For non-spatial transcriptomic baseline comparison, we performed standard Louvain clustering on normalized Stereo-seq data. Batch effects were mitigated using Harmony (v0.1.1) on each group sections. A SNN graph (*n_neighbors* = 30) was constructed from corrected embeddings, and clusters were identified at a resolution of 0.8. (6) GraphST: Each sample was processed as an *AnnData* object with unique feature identifiers. Spatially informed embeddings were learned using *datatype* = “Stereo” setting. Spatial domains were identified using the built-in clustering function (via *mclust*) with the cluster number pre-set to match anatomical expectations. For visualization, spatial coordinates were plotted using Scanpy, with the y-axis inverted to preserve the anatomical orientation of the Sect.  (7) BANKSY: To incorporate local spatial context, spatial neighborhood features were computed with computeBanksy (*compute_agf* = TRUE, *k_geom* = 16). Demensionality reduction was performed via *runBanksyPCA()* with ***λ*** = 0.8 and *npcs* = 18. BANKSY embedding was subsequently integrated across sections using Harmony with T_names as the batch variable to account for inter-sample variation before UMAP visualization.

### Benchmarking of clustering methods against manual cortical layer annotations

To benchmark different spatial clustering methods for cortical layer identification, manually annotated cortical layers were used as the reference. Clustering results from multiple methods were collected in a unified object, and the similarity between each clustering result and the manual layer labels was quantified for each section using the ARI. ARI values were computed independently for all sections and methods, and were used to compare the ability of different approaches to recover cortical laminar structure. Higher ARI values indicated better agreement with manual layer annotations.

### Sensitivity analysis of BayesSpace clustering parameters

A sensitivity analysis was performed to assess the effect of cluster number (n_clu) on BayesSpace results. BayesSpace clustering was run across a range of n_clu values, and clustering similarity between parameter settings was quantified using the ARI. Pairwise ARI values were visualized as a heatmap to evaluate clustering stability across different n_clu values. For each n_clu, concordance between BayesSpace clusters and manually annotated cortical layers was further assessed using ARI, and cluster specificity was measured using purity. These analyses were used to identify a stable and biologically interpretable range of cluster numbers for downstream analyses.

### Determination of optimal cluster number in unsupervised clustering

To identify the optimal number of clusters (*k*) for BayesSpace, we employed a scalable discrepancy-based internal validation metric, *fasthplus*, to evaluate clustering performance across varying *k* values. In unsupervised scenarios lacking external ground-truth labels (e.g., adjusted Rand index, ARI), internal validation metrics such as cluster compactness (e.g., within-cluster sum of squares, WCSS) or separation (e.g., Silhouette index) are typically used. The *H+* metric in *fasthplus* (v1.0) quantifies rank-based discrepancies in pairwise distances within predicted clusters, providing a robust measure of clustering stability. We computed *H+* scores for candidate *k* values and generated a 1-*H+* curve. The second inflection point of this curve-where the *H+* score plateaued-was selected as the optimal *k*. This data-driven approach minimizes subjectivity in cluster number selection.

### Visualization of Allen Brain Atlas ISH data

For visualization of spatial expression patterns in the Allen Human Brain Atlas, target genes were queried in the Allen Brain Atlas (ABA) human in situ hybridization (ISH) database through the Allen Human Brain Atlas website (https://human.brain-map.org/ish/search). For each gene, available ISH image series were manually inspected. Sections were selected for display if they contained the cerebral cortex with clearly identifiable cortical lamination, showed interpretable staining signals, and had no major tissue damage, severe staining artifacts, or image-quality issues that could affect visual assessment. When multiple sections met these criteria, one representative section was selected for qualitative visualization. These ABA ISH images were used only to illustrate the spatial expression patterns of selected genes and were not included in the quantitative analyses.

### Stereo-seq cell type annotation

1. SpatialID analysis was performed following the official instructions provided in the SpatialID repository. For label transfer from the reference dataset to the spatial transcriptomic dataset, the parameters were set as follows: *pca_dim* = 200, *k_graph* = 30, *edge_weight* = TRUE, *epochs* = 200, *w_cls* = 20, *w_dae* = 1, and *w_gae* = 1.
2. Cell2location analysis was conducted using the implementation provided in the Cell2location repository. Model training was performed with *max_epochs* = 300, *batch_size* = 5,000, and *train_size* = 1.
3. Tangram analysis was carried out using the code available in the Tangram repository. For the *map_cells_to_space* function, the parameters were set to *mode* = “clusters”, *density_prior* = “rna_count_based”, and *num_epochs* = 1,000.
4. DestVI analysis was performed using the implementation in scvi-tools. The parameter *max_epochs* = 300 was used for training both the snRNA-seq model and the spatial model.
5. RCTD analysis was conducted using the spacexr repository (v2.0.0). During reference data processing, the parameters were set to *n_max_cells* = 10,000 and *min_UMI* = 50.
6. SPOTlight analysis was performed using SPOTlight (v1.2.0) from Bioconductor. For the *getTopHVGs* function, *n* = 1,000 was used to select the top 1,000 highly variable genes. In the SPOTlight deconvolution step, *weight_id* = “mean.AUC” was used as the weighting parameter.
7. Seurat-based label transfer was performed following the Seurat v4 reference mapping workflow. In the *FindTransferAnchors* function, the parameters were set to *dims* = 1:20, *normalization.method* = “LogNormalize”, and *reference.reduction* = “pca”.
8. SPANN analysis was implemented using SPANN-torch according to the developer’s instructions. The hyperparameters were set as follows: *learning rate* = 2e-4, *lambda_spa* = 0.001, *lambda_cd* = 0.001, *lambda_nb* = 10, *maxiter* = 5,000, *maxiter1* = 2,000, *maxiter2* = 4,000, and *maxiter3* = 40,000.

### Accuracy scoring system

The accuracy scoring system was calculated by first determining the average PPMCC (Pearson product–moment correlation coefficient), KL (Kullback–Leibler), SSIM (structural similarity) and TYPE (the number of identified cell types) values for all cell types predicted by each transfer method. Subsequently, the PPMCC, SSIM, and TYPE were ranked in ascending order, while the KL was ranked in descending order. The ranks of the four metrics were then summed and normalized to yield an ASS score ranging from 0 to 1. The ASS score was calculated as follows:

$$\mathrm{ASS}={\mathrm{rank}}_{\mathrm{PPMCC}}+{\mathrm{rank}}_{\mathrm{KL}}+{\mathrm{rank}}_{\mathrm{SSIM}}+{\mathrm{rank}}_{\mathrm{TYPE}\cdot}$$

### Cell subtype identification

Cellular subtypes were identified through an integrated computational pipeline. Briefly, cell populations were first extracted from Stereo-seq cellbin data using the *subset()* function based on preliminary cell-type annotations. The isolated data were normalized using SCTransform for variance stabilization, followed by batch effect correction via Harmony integration with default parameters. A shared nearest neighbor (SNN) graph was constructed using the top 30 principal components (*FindNeighbors*, dims = 1:30), and subclustering was performed with the Louvain algorithm (*FindClusters*). After evaluating resolutions ranging from 0.1 to 1.0, we selected res = 0.3 as the optimal parameter based on silhouette scores (> 0.6) and concordance with canonical marker gene expression patterns (Wilcoxon rank-sum test, FDR < 0.01). This resolution balanced cluster granularity and biological interpretability, yielding X distinct subtypes for downstream analyses.

### Differential expression analysis

Differential gene expression analysis was conducted using the *FindAllMarkers* function in the Seurat package (v4.3.0) to compare transcriptional profiles across distinct cell types and group sections. The Benjamini-Hochberg (BH) method was applied to control the false discovery rate (FDR). Genes with log_2_(fold change) > 0.25 and an adjusted_FDR < 0.05 were classified as differentially expressed genes (DEGs).

### XGBoost-based label transfer

We used XGBoost [18] to evaluate label correspondence between datasets. After selecting shared informative genes from the reference and comparison datasets, a multiclass XGBoost classifier was trained on the reference dataset and used to predict labels in the comparison dataset. Prediction consistency was assessed using confusion matrices, ARI, and normalized conditional entropy (NCE).

### Gene counts downsampling analysis

To mitigate the potential impact of variations in gene capture efficiency on clustering outcomes, we performed counts downsampling on Group I sections using the *SampleUMI()* function. Since all Group III chips exhibited counts exceeding 10,000, we implemented downsampling with parameters set to *max.umi* = 10,000 and *upsample* = FALSE. Following this normalization procedure, the processed data were subsequently re-analyzed for clustering and systematically compared with the Group III sections.

### Quantification of laminar disorganization

To quantify laminar disorganization, we used AD-Ls as a structural reference and assessed the distribution of Bayes20 clusters within each layer. For each section, we calculated the Shannon entropy of Bayes20 clusters within each layer to measure domain mixing, and layer purity as the proportion of spots assigned to the dominant Bayes20 cluster. Section-level scores were derived using spot-number-weighted averages. Group differences were evaluated using two-sided Wilcoxon rank-sum tests with Benjamini-Hochberg FDR correction for multiple comparisons.

### Functional enrichment analysis

Gene Ontology (GO) enrichment analysis for biological processes (BP) was conducted on the identified DEGs using the clusterProfiler software package (v4.2.2). Significantly enriched GO terms were defined as those with an FDR < 0.05.

### Disease ontology enrichment analysis

Gene symbols of interest were converted into Entrez IDs, and over-representation analysis was conducted against the Disease Ontology database using the enrichDO function in the clusterProfiler package. The background gene set was defined as all expressed genes detected in the dataset. Enriched disease terms with an adjusted *P* value < 0.05 after Benjamini-Hochberg correction were considered statistically significant. The top enriched DO terms were visualized using a lollipop plot.

## Results

### Anatomy-defined parcellation of human cortical layers

**Fig. 1:**
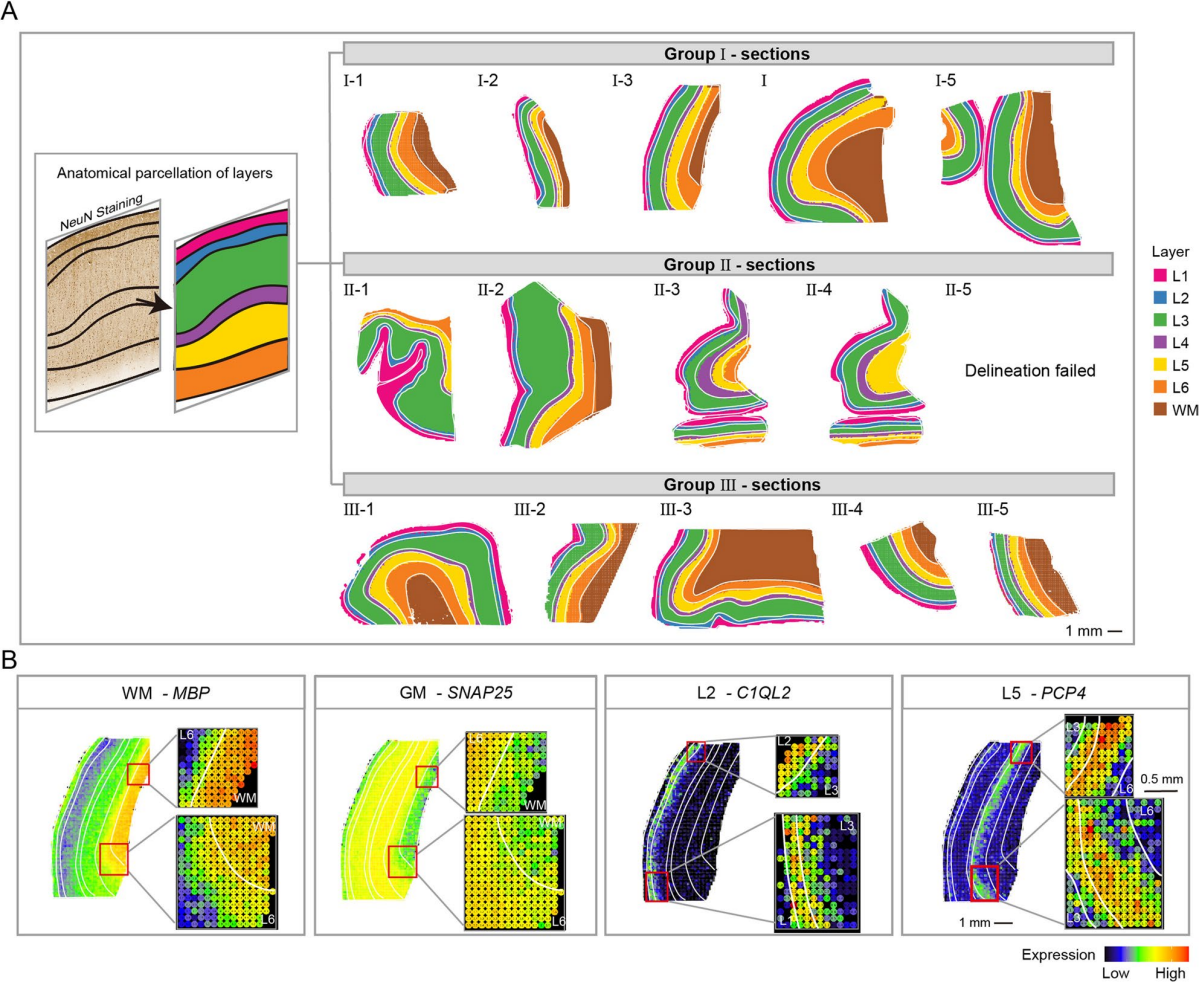
Anatomy-defined layer parcellation of the human cerebral cortex. **A** Layer parcellation of Stereo-seq sections based on anatomy-defined layer parcellation (AD-Ls) (see Methods). **B** Spatial expression heatmaps of canonical marker genes. White lines indicate the boundaries defined by AD-Ls. The left panel shows the spatial expression of each gene (scale bar, 1 mm). The right panel presents a magnified view of the red-outlined region, with black numbers indicating gene expression levels in each Bin200 spot (scale bar, 0.5 mm)

To accurately delineate cortical layer boundaries, we performed high-resolution Stereo-seq on 15 human cortical sections from 14 surgical donors, covering four cortical lobes (Supplementary Material 2: Fig. S1A, B). Using neuronal nuclear protein (NeuN) staining, experienced neuroanatomists identified distinct cortical layers across these sections (Fig. 1A). Notably, due to poor staining quality, section II-5 could not be reliably assigned to classical cortical layers based on established anatomical criteria, highlighting a limitation of anatomy-defined layer parcellation (AD-Ls). Based on morphology and section quality, we classified the sections into three groups (Groups I–III, five sections each) to facilitate comparative analysis (Fig. 1A).

We further validated the accuracy of AD-Ls using classical layer-specific marker genes [9]. Their spatial expression largely aligned with the anatomically identified layers (Supplementary Material 2: Fig. S1C). However, discrepancies persisted; for instance, the gray matter (GM) region adjacent to white matter (WM) showed high expression of the WM-enriched marker *MBP*, but low expression of the GM-enriched marker *SNAP25* (Fig. 1B) [9]. Moreover, some marker genes traditionally associated with specific layers, such as *C1QL2* for Layer (L) 2 [9], and *PCP4* for L5 [5, 9], were expressed across multiple layers or transitional zones (Fig. 1B; Supplementary Material 2: Fig. S1D), indicating limited precision of the anatomy-based boundaries when assessed by gene expression patterns. These results indicate that the layer boundaries identified by AD-Ls have limited accuracy relative to the spatial expression patterns of classical layer markers.

### Gene-expression-defined parcellation of human cortical layers

**Fig. 2:**
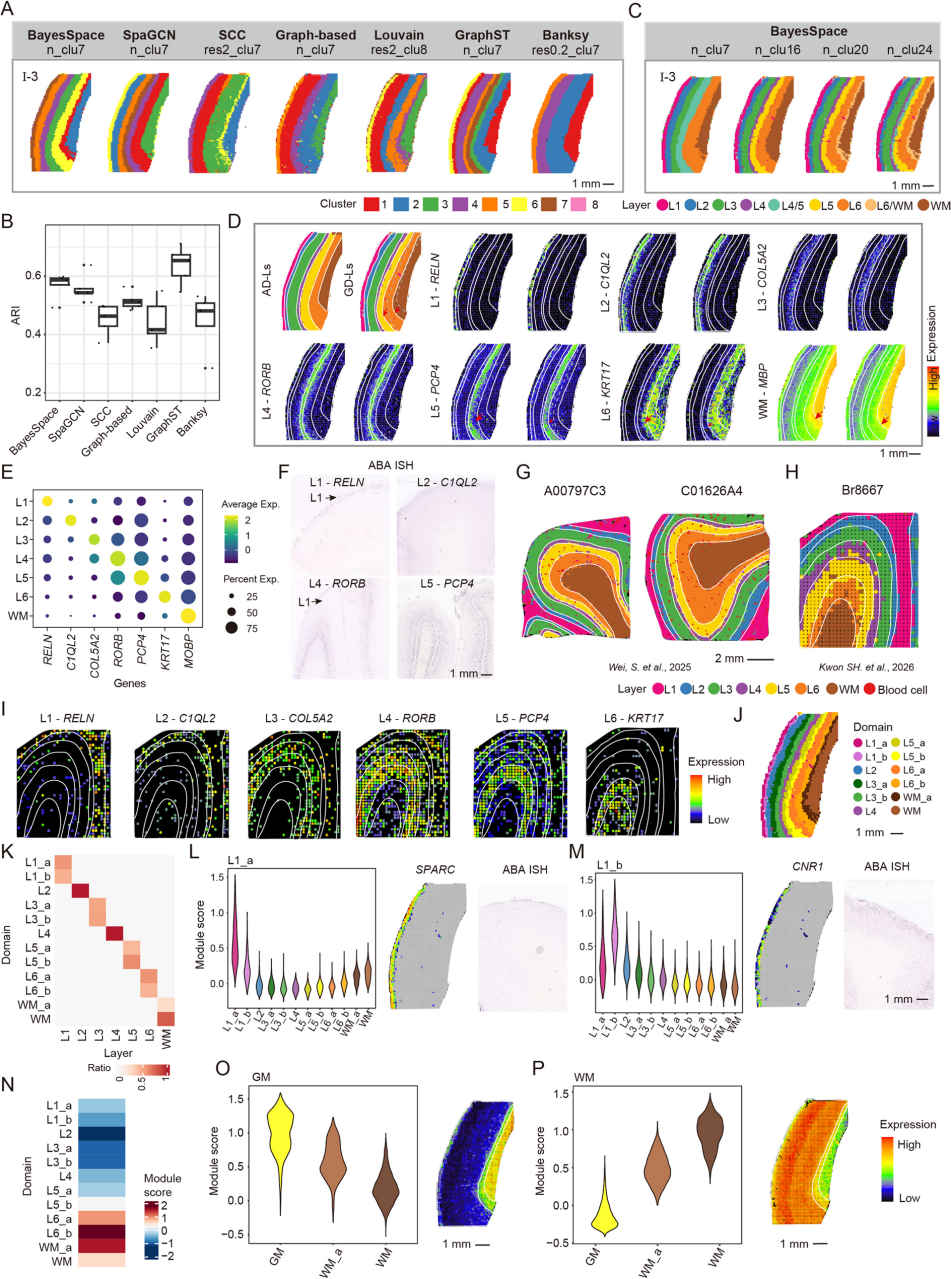
Gene expression-defined cortical layer parcellation in the human cerebral cortex. **A** Cortical layer parcellation using seven distinct clustering methods based on Group Ⅰ-3 Bin200 Stereo-seq data (see Methods). Scale bar: 1 mm. **B** Clustering accuracy across Group Ⅰ sections evaluated by the Adjusted Rand Index (ARI), with higher values indicating better performance. **C** Uniform manifold approximation and projection (UMAP) visualizations of BayesSpace clustering results at different resolutions. Scale bar: 1 mm. **D** Comparison of AD-Ls and GD-Ls. Left: AD-L boundaries (white lines). Right: GD-L boundaries (white lines). Red arrows indicate boundary adjustments from AD-Ls to GD-Ls. Scale bar: 1 mm. **E** Layer-specific gene expression in Group Ⅰ. **F** Representative layer marker genes shown by *in situ* hybridization images from the Allen Brain Atlas (ABA ISH).(**G-H**) Laminar identification of the healthy human cerebral cortex using GD-Ls in Stereo-seq [25] (**G**) and 10x Visium [22] (**H**). **I** Heatmap showing layer marker gene expression in the brain section shown in panel **H**. **J** Sublayer-like domain parcellation identified using GD-Ls with Bin200 data. Scale bar: 1 mm. **K** Heatmap showing the proportion of spots assigned to each domain within each cortical layer. **L**, **M** Module scores of domain-specific genes across cortical domains, along with representative domain marker genes enriched in each domain (gray indicates other domains) and their corresponding ABA ISH images. **N** Heatmap displaying module scores of genes specifically expressed in L6b neurons from snRNA-seq data across all domains. **O**, **P** Violin plots and spatial heatmaps showing the expression of gray matter (GM) and white matter (WM) marker genes across GM, WM, and transitional WM_a domains

Given the critical importance of accurate layer identification for cortical research, we leveraged the molecular resolution of gene expression profiles to improve layer parcellation accuracy. Based on preliminary analyses of Group I sections, we systematically optimized parameters including bin size and clustering methodology. To determine an appropriate bin size, and following prior studies of the human cerebral cortex [9], we applied BayesSpace clustering (n_clu = 16) across multiple bin sizes (Bin50, Bin100, Bin150, Bin200, Bin300) (Supplementary Material 3: Fig. S2A; Supplementary Material 1: Table S2). Quantitative evaluation showed that intermediate bin sizes (Bin100 to Bin200) achieved the best overall balance between concordance with manual layers, cluster specificity, and spatial continuity (see Methods). Bin50 appeared too sparse, whereas Bin300 showed evidence of increased smoothing. We therefore considered Bin100 to Bin200 as a robust parameter range and selected Bin200 for downstream analyses (Supplementary Material 3: Fig. S2B, C). Regarding the clustering methods, we benchmarked seven clustering methodologies, including batch-corrected BayesSpace, SpaGCN, spatially-constrained clustering (SCC), graph-based clustering, the Louvain algorithm, GraphST and Banksy, using approximately seven clusters (Fig. 2A, Supplementary Material 3: Fig. S2D) [11–17]. Among these methods, BayesSpace exhibited superior layer discrimination and resolved discrete cortical domains with enhanced granularity across all sections. Although GraphST also performed relatively well, it occasionally merged distinct cortical layers in some sections and was less convenient for joint batch-corrected clustering across donors than BayesSpace (Fig. 2A, B). Consequently, we selected BayesSpace for subsequent analyses. We next performed a sensitivity analysis to determine the appropriate BayesSpace cluster number for laminar delineation. Clustering solutions remained relatively stable within an intermediate range of cluster numbers, particularly n_clu = 13–20, whereas lower values tended to under-segment cortical laminae and higher values led to increasing subdivision (Supplementary Material 4: Fig. S3A-C). Together with the Fastplus (H+) analysis (Supplementary Material 4: Fig. S3D) [19], these results supported 13–20 as a robust range of n_clu values. We then examined white matter (WM) and gray matter (GM) annotation at representative resolutions (n_clu = 7, 16, 20, 24) using layer-specific marker gene expression (Fig. 2C; Supplementary Material 4: Fig. S3E-G). The n_clu = 7 solution was too coarse to reliably distinguish L4/5 or remove low-quality spots, whereas n_clu = 24 produced finer subdivisions, including transitional zones at the L6/WM interface and blood cell-enriched clusters. By contrast, solutions with n_clu values of 16–20 provided the best balance between laminar resolution, spatial interpretability, and biological relevance. These results supported n_clu = 16–20 as a robust parameter range for GD-L parcellation. Therefore, we used Bin200, BayesSpace and representative cluster numbers within this interval (n_clu = 16–20) to define gene-expression-defined layers (GD-Ls) in subsequent analyses. Using these optimal parameters, BayesSpace was employed for layer parcellation based on gene expression profiles. BayesSpace facilitated laminar identification across all sections after batch effect correction. More than half of the sections showed robust consistency of laminar boundaries with AD-Ls, including sections Ⅰ-1, Ⅰ-2, and Ⅰ-5 (Supplementary Material 5: Fig. S4A). However, certain boundaries, like those between L5/6 and L6/WM in sections Ⅰ-3 and Ⅰ-4, were accurately refined (Fig. 2D; Supplementary Material 5: Fig. S4B). These corrected layer boundaries were further validated using classical layer-specific markers, including *RELN* for L1, *C1QL2* for L2, *COL5A2* for L3, *RORB* for L4, *PCP4* for L5, *KRT17* for L6, and *MBP* for WM (Fig. 2D, E; Supplementary Material 5: Fig. S4B, C). Among these, *RELN*, *C1QL2*, *RORB*, and *PCP4* were further validated as layer-enriched genes using in situ hybridization data from the Allen Brain Atlas (ABA ISH) (Fig. 2F). To further evaluate the cross-condition generalizability of GD-Ls beyond surgical specimens, we applied the framework to independent healthy postmortem human cortical ST datasets. The results indicated that GD-Ls stably resolved cortical layers in two Stereo-seq datasets from individuals without neurological disease, faithfully recapitulating the laminar expression patterns of canonical marker genes (Fig. 2G; Supplementary Material 5: Fig. S4D-G). Similar results were obtained in healthy postmortem human cortical sections profiled by 10x Visium (Fig. 2H, I; Supplementary Material 5: Fig. S4H), demonstrating that GD-Ls capture intrinsic architectural features of the human cortex across both surgical and postmortem samples and across different ST platforms. Furthermore, except for L2 and L4 in GM, several classical layers could be further resolved into two molecularly distinct sublayer-like domains (Fig. 2J, K; Supplementary Material 6: Fig. S5A). Domain-enriched marker genes were characterized (Supplementary Material 1: Table S3), and enrichment scoring showed spatially restricted expression patterns, including *SPARC* in L1_a, *CNR1* in L1_b, *NEFH*/*NEFM*/*NEFL* in L3_b, and *NR4A2*/*SMIM32* in L6_a. These observations were further supported by ABA ISH data, in which *SPARC*, *CNR1*, and *NEFH* displayed concordant laminar enrichment patterns (Fig. 2L, M; Supplementary Material 6: Fig. S5B, C). These domains were sufficiently stable to be identified across other healthy cortical datasets (Supplementary Material 6: Fig. S5D). These results suggest that the inferred sublayer-like domains represent reproducible molecular subdivisions within the cortical ribbon, although their functional significance remains to be established. Previous studies have identified L6b as a sublayer of L6, enriched in L6b neurons [20, 21]. Here, we found that the L6b neuronal marker genes were highly expressed in the L6_b domain (Fig. 2N), and the L6b marker *CCN2* was specifically expressed in this domain (Supplementary Material 6: Fig. S5B, C). These findings suggest that the L6_b domain corresponds to the known L6b sublayer in the human cerebral cortex. We also observed a WM-associated transitional domain (WM_a) at the GM-WM interface, characterized by co-expression of canonical GM and WM markers (Fig. 2O, P; Supplementary Material 6: Fig. S5E). This pattern is consistent with a superficial white matter (SWM)-like transitional zone [22–24] and may aid future identification of the GM-WM junction.

Overall, the GD-Ls not only enable precise parcellation of the human cerebral cortex with improved accuracy in defining interlayer boundaries, thereby complementing or refining AD-Ls, but also delineate sublayers-like domains marked by distinct expression markers.

### Accurate distribution of cortical cell types through GD-Ls

**Fig. 3:**
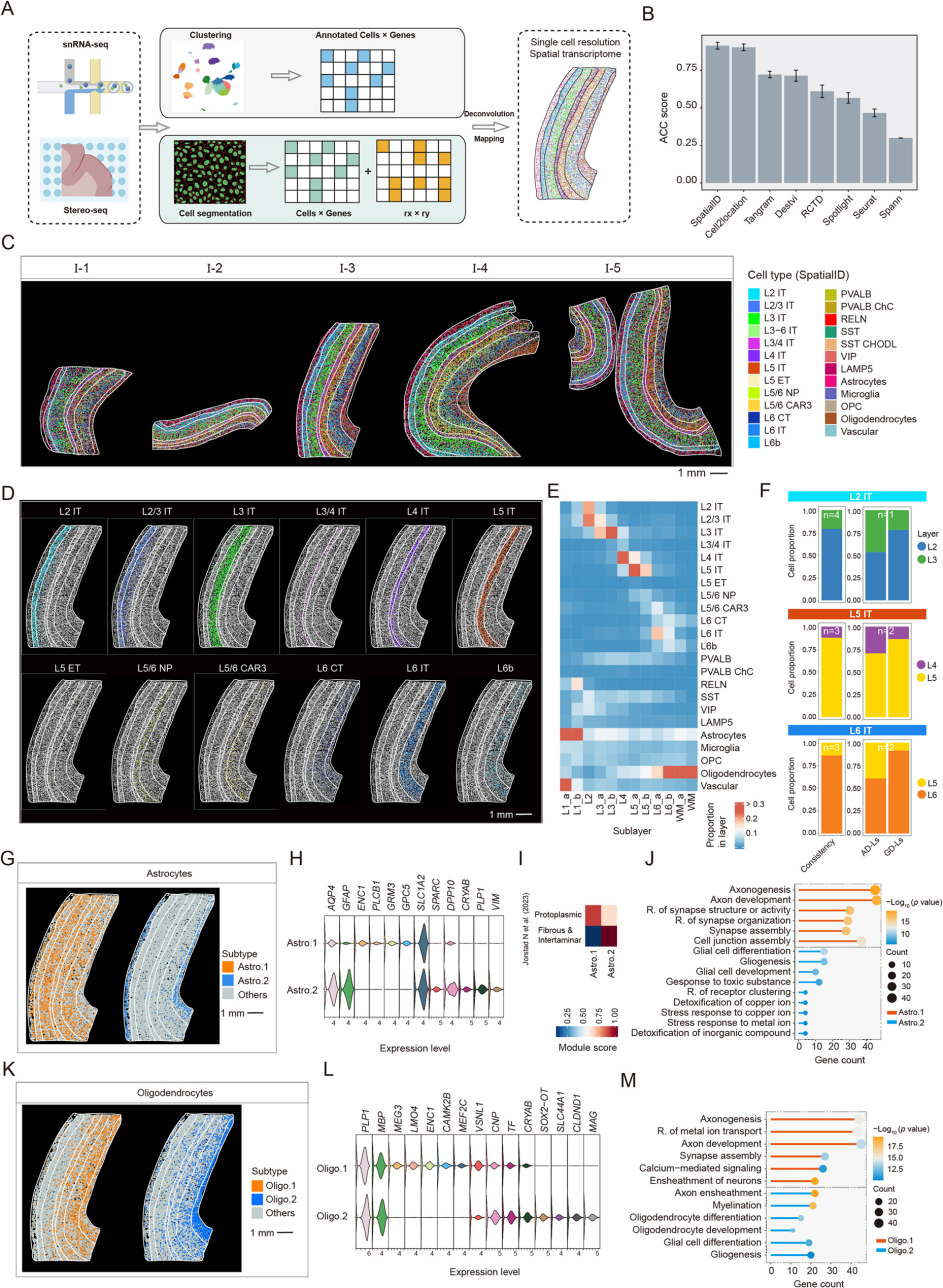
Cell type distribution in the human cortex. **A **Single-cell segmentation and cell type annotation workflow. **B** Accuracy scores of the various algorithms based on the accuracy scoring system. **C** Spatial distribution of cell types predicted by SpatialID, with colors indicating distinct cell types. **D** Spatial distribution of excitatory neurons with layer-specific patterns. **E** Heatmap showing the proportions of each cell type across different sublayer-like domains in Group Ⅰ. **F** Bar plot showing the layer proportions of three IT neuron types in Group I across AD-Ls and GD-Ls. "Consistency" indicates neurons from sections with concordant GD-Ls and AD-Ls parcellation. Sample numbers are labeled in each panel: for L2 IT, n = 1 refers to section I-3 only, whereas the other panels include sections I-3 and I-4. **G**, **K** Spatial distribution of astrocyte subtypes (**G**) and oligodendrocyte subtypes (**K**). **H**, **L** Violin plots showing the expression of markers for astrocyte subtypes (**H**) and oligodendrocyte subtypes (**L**). I Heatmap of module scores for astrocyte subtype-specific gene sets reported by Jorstad et al., 2023 [30]. **J**, **M** Lollipop plots showing GO enrichment analysis of marker genes for astrocyte subtypes (**J**) and oligodendrocyte subtypes (**M**)

To determine the spatial distribution of cortical cell types, we first segmented cells from single-stranded DNA (ssDNA) staining images acquired by Stereo-seq using the watershed algorithm [7]. We then transferred cell annotations from the reported snRNA-seq data [10] to the segmented cells, evaluating eight distinct transfer methods (Fig. 3A; Supplementary Material 7: Fig. S6A-C). Among these, SpatialID (27) and Cell2location (28) achieved the highest accuracy scores according to the accuracy scoring system (ASS) (Fig. 3B; Supplementary Material 5: Table S4) [29]. SpatialID was selected for subsequent analysis because it produced a biologically plausible spatial distribution and cell type composition (Fig. 3C; Supplementary Material 7: Fig. S6D,E), while also showing strong concordance with the snRNA-seq cell types (Supplementary Material 7: Fig. S6F-G). The results revealed that excitatory neurons exhibited distinct laminar distributions (Fig. 3D, E; Supplementary Material 7: Fig. S6H; Fig. S7). For instance, L2 IT neurons were confined to L2, whereas L5/6 IT Car3 neurons localized to the boundary between L5 and L6. Among layer-enriched IT neuron subtypes, the proportions of cells distributed in the corresponding layers after GD-Ls boundary adjustment were similar to those in sections showing concordance between AD-Ls and GD-Ls, consistent with the utility of GD-Ls for boundary delineation (Fig. 3F). Excitatory non-IT neurons primarily occupied deeper layers, with L5 ET neurons concentrated in L5 (Fig. 3D; Supplementary Material 7: Fig. S6H). L6b neurons exhibited a sublayer distribution, predominantly in L6b and WM_a (Fig. 3D, E; Supplementary Material 7: Fig. S6J), which aligned with the spatial expression patterns of the L6b neuronal marker gene *CCN2* (Fig. 2N; Supplementary Material 6: Fig. S5C). Similarly, specific inhibitory neuron types displayed layer- and domain-restricted localization: RELN neurons were enriched in L1, particularly in L1_b, and their distribution boundaries corresponded closely to those defined by GD-Ls, whereas VIP neurons were mainly found in L2 and L3 (Fig. 3E; Supplementary Material 7: Fig. S6I). Other inhibitory neurons, such as PVALB neurons, were more diffusely distributed (Supplementary Material 7: Fig. S6I). The WM_a domain, located at the GM-WM boundary, contained a cellular composition intermediate between L6_b and WM, including both L6 CT neurons and L6b neurons. WM_a also harbored a substantial population of glial cells, including oligodendrocytes and astrocytes (Fig. 3E). Non-neuronal cell densities also varied across cortical layers: oligodendrocytes were denser in L6 and WM, vascular cells were concentrated in L1_a, and astrocytes showed a diffuse distribution with higher density in L1, whereas microglia and OPCs were more sparsely distributed (Fig. 3E; Supplementary Material 7: Fig. S6I).

To investigate whether non-neuronal subtypes exhibited laminar specificity, we identified subtypes within non-neuronal cell types. Astrocytes were classified into two subtypes with distinct spatial distributions, gene expression profiles, and biological functions (Fig. 3G-J). Astro.1 was diffusely distributed, specifically expressing genes such as *ENC1*, *PLCB1*, and *GRM3*, and was enriched in pathways related to axonogenesis, axon development, and synapse assembly. In contrast, Astro.2 was restricted to L1 and WM, expressed high levels of *PLP1* and *VIM*, and was enriched in pathways associated with gliogenesis, glial cell differentiation, and response to ion. These two subtypes corresponded to previously described protoplasmic and fibrous/interlaminar astrocytes, respectively (Fig. 3I) [30]. Similarly, Oligo.1 was diffusely distributed in deep layers and WM, expressed *MEG3*, *LMO4*, and *ENC1*, and functioned in axonogenesis and synaptic processes. Oligo.2, which specifically expressed *SOX2-OT*, *SLC44A1*, and *CLDND1*, was primarily localized to WM and was enriched in pathways related to myelination, oligodendrocyte development and differentiation (Fig. 3K-M).

These results demonstrate that GD-Ls effectively delineate the spatial distribution patterns of diverse cell types and subtypes, further supporting the accuracy of GD-Ls.

### GD-Ls accurately delineate curved cortical layers

**Fig. 4:**
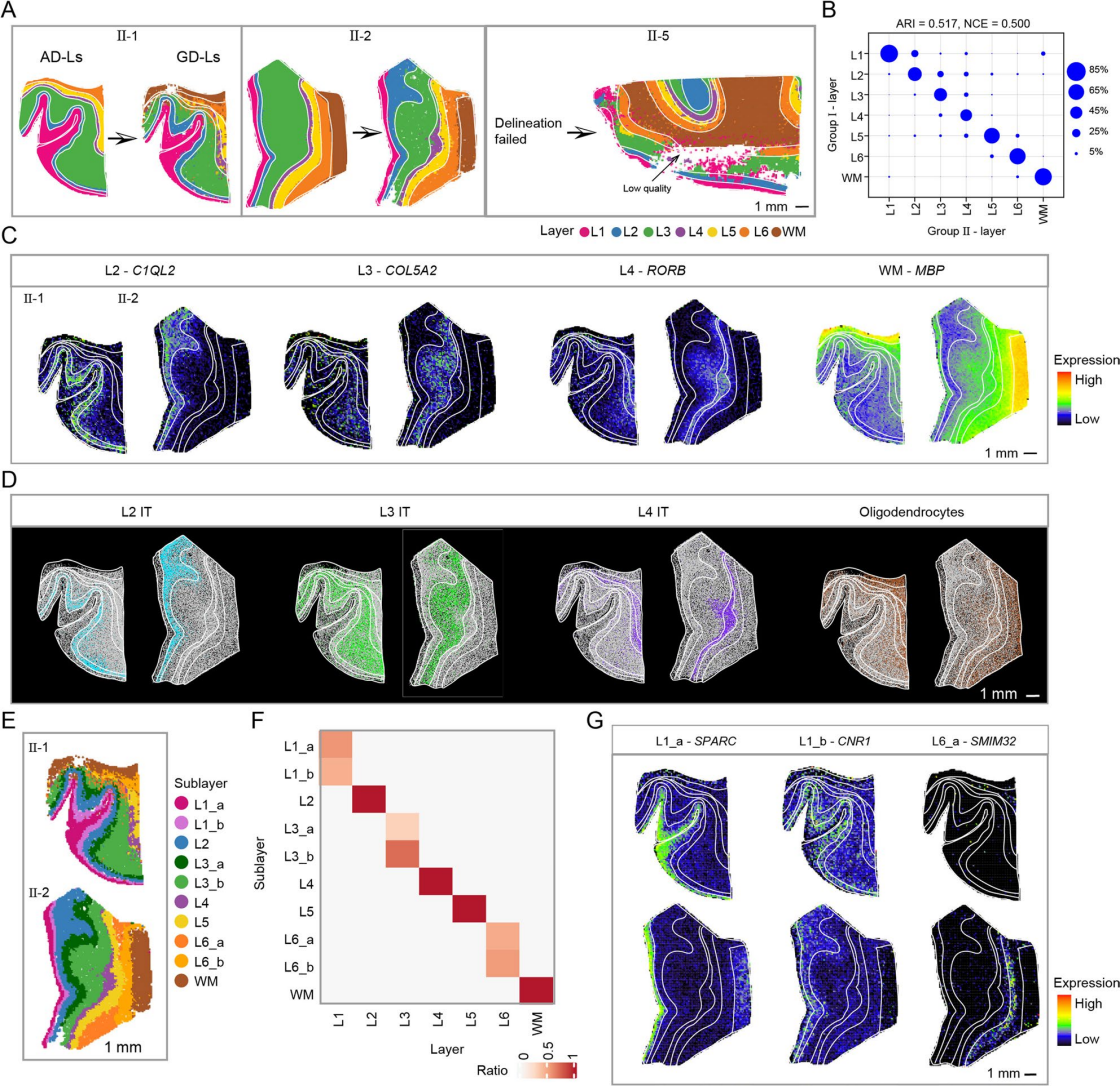
Gene expression-defined layer parcellation of curved cortical sections. **A** Spatial UMAP visualization showing cortical layer identification by AD-Ls and GD-Ls. **B** Dot plot showing the correlation of layer profiles between Group I and Group II. **C**, **D** Spatial maps illustrating the concordance between GD-Ls and canonical layer marker genes (**C**) and layer-enriched cell type distributions (**D**). White lines indicate the boundaries defined by GD-Ls. **E** UMAP visualization of sublayer-like domains. **F** Heatmap showing the distribution of sublayer-like domains within each layer. **G** Spatial expression patterns of marker genes for sublayer-like domains

We next investigated whether GD-Ls could precisely delineate curved cortical layers in Group Ⅱ sections. Given the structural complexity of these curved layers, we first partitioned the sections into 20 clusters using BayesSpace (Supplementary Material 9: Fig. S8A). Applying the GD-Ls parcellation method, we clearly defined the interlayer boundaries, including in section II-5, which could not be resolved using AD-Ls (Fig. 4A; Supplementary Material 9: Fig. S8A-E). Each layer in Group Ⅱ sections showed high correlation with the corresponding layers in Group Ⅰ sections (Fig. 4B). Furthermore, spatial expression patterns of layer-enriched markers and the distributions of layer-specific cell types confirmed the accuracy of GD-Ls parcellation in Group Ⅱ (Fig. 4C, D; Supplementary Material 9: Fig. S8F-G). Because section II-5 had poor ssDNA image quality, no cell bin-level data were obtained, and cell bin-level analyses were therefore not performed. GD-Ls also reliably identified sublayer-like domains throughout Group Ⅱ sections (Fig. 4E, F; Supplementary Material 9: Fig. S8H, I), which was supported by sublayer-specific marker expression (Fig. 4G). These results demonstrate that GD-Ls can precisely parcellate the curved cortex.

Intriguingly, the layers in sections Ⅱ-3 and Ⅱ-4, derived from histologically normal peri-tumoral tissue, showed a poorly defined boundary between L4 and L5 (Supplementary Material 9: Fig. S8E). In these sections, the L5 marker *PCP4* was also expressed in L6, a pattern not observed in other sections from Groups Ⅰ and Ⅱ (Supplementary Material 5: Fig. S4C; Fig. S8F). Additionally, the distribution of many IT neurons in Ⅱ-3 and Ⅱ-4 sections appeared disorganized relative to Group Ⅰ. Both L4 IT and L5 IT neurons were co-localized within the same cortical region, while L3 IT neurons extended into L4/5. Oligodendrocytes were also densely packed in L4/5 (Supplementary Material 9: Fig. S8G; Fig. S8J). These findings suggest a potential underlying pathology despite the normal histological appearance, indicating that GD-Ls can sensitively detect molecular pathological changes.

### GD-Ls accurately delineate cortical layers across species

**Fig. 5:**
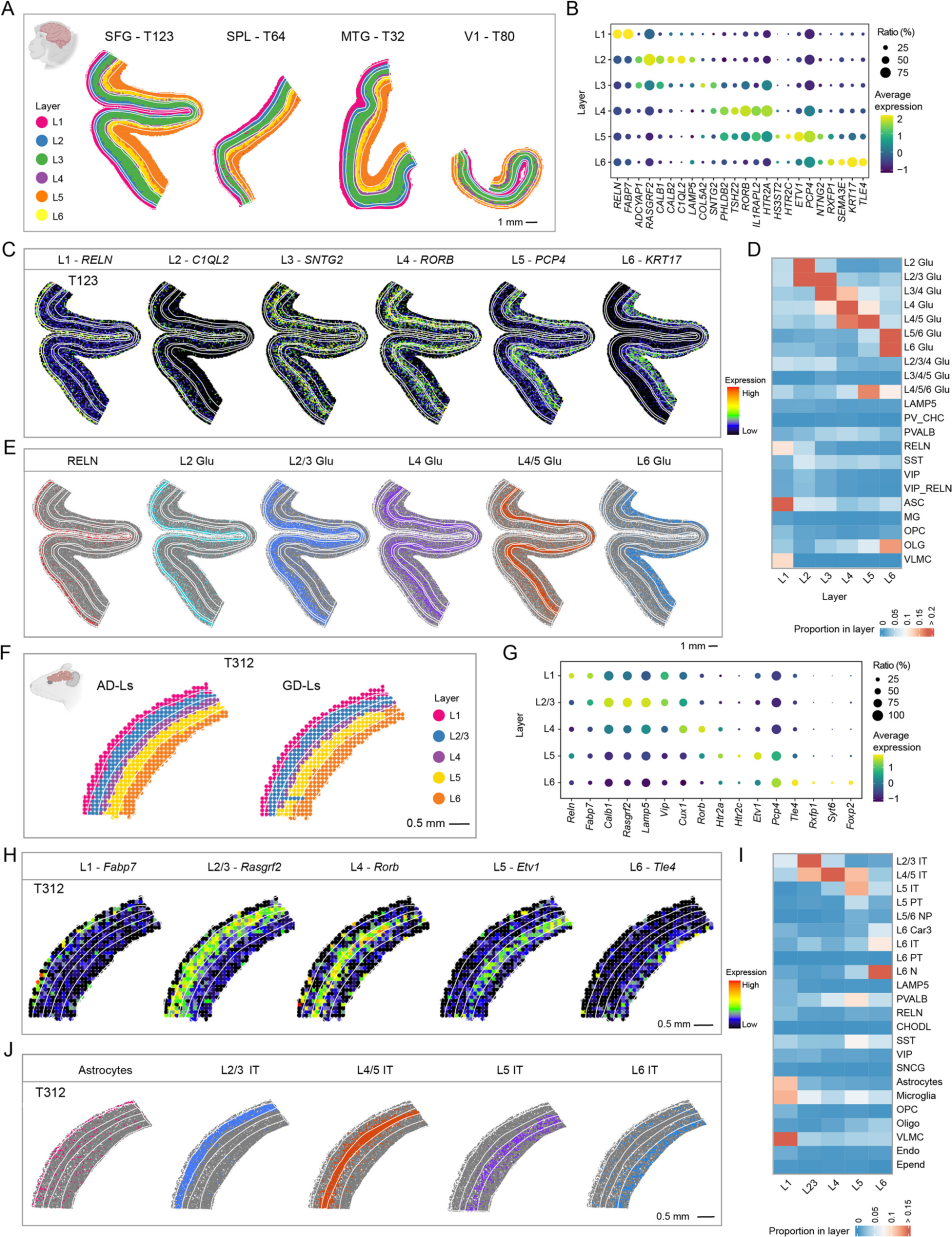
Identification of cortical layers in macaque and mouse using GD-Ls. **A** Spatial UMAP visualization of GD-Ls (color) in the macaque cortex, with white lines indicating AD-Ls. Scale bar: 1 mm. **B** Dot plot showing the expression of layer-specific marker genes across macaque cortical layers. **C**, **E** Spatial maps displaying macaque layer marker gene expression (**C**) and cell-type distributions (**E**). White lines indicate the boundaries defined by GD-Ls. **D** Heatmap showing the proportion of each cell type across macaque cortical layers. **F** Spatial UMAP visualization illustrating the consistency between GD-Ls and AD-Ls in the mouse cortex, with the white lines indicating the boundaries defined by AD-Ls. Scale bar: 0.5 mm. **G** Dot plot showing the expression of layer marker genes across mouse cortical layers. **H**, **J** Spatial maps displaying mouse layer marker gene expression (**H**) and cell-type distributions (**J**). White lines indicate the boundaries defined by GD-Ls. Scale bar: 0.5 mm. **I** Heatmap showing the proportion of each cell type across mouse cortical layers

To determine whether the cortices of other mammals could also be parcellated with GD-Ls, we analyzed published Stereo-seq data from macaque [6] and mouse [7] cortices. Four macaque cortical sections, representing the superior frontal gyrus (SFG, T123), superior parietal lobule (SPL, T64), middle temporal gyrus (MTG, T32), and primary visual cortex (V1, T80), were analyzed using GD-Ls with optimal parameters (Bin200, BayesSpace, n_clu = 16) (Supplementary Material 10: Fig. S9A, B). Using macaque-specific layer-enriched markers, six cortical layers were identified by GD-Ls (Fig. 5A, B; Supplementary Material 10: Fig. S9C). The layers identified by GD-Ls were more consistent with layers demarcated by AD-Ls across whole macaque cortical sections than in human cortical sections (Fig. 5A). Both macaque and human cortices shared conserved layer-enriched genes, whose expression aligned with GD-Ls-defined layers (Fig. 5B, C; Supplementary Material 10: Fig. S9D). Nevertheless, we also detected macaque-specific layer markers, including *SNTG2* (enriched in L3) and *TLE4* (enriched in L6), consistent with previous studies (Fig. 5B, C; Supplementary Material 10: Fig. S9D) [6, 31]. Cell-type distributions in the macaque cortex resembled those in humans, with excitatory neurons displaying distinct layer-specific organization (Fig. 5D, E; Supplementary Material 10: Fig. S9E-G).

Similarly, we extracted three cortical sections from whole coronal mouse brain sections (T302, T312, and T322) (Supplementary Material 11: Fig. S10A) and applied the GD-Ls method for layer identification. We again observed strong concordance between GD-Ls and AD-Ls (Fig. 5F-G; Supplementary Material 11: Fig. S10B-D). In contrast to the macaque cortex, however, mouse cortical layer-enriched genes diverged more substantially from those in the human cortex (Fig. 5H; Supplementary Material 11: Fig. S10E). Human and macaque layer-specific markers such as *RELN*, *C1QL2*, *COL5A2*, and *KRT17* lacked clearly enriched laminar expression patterns in mouse cortex (Supplementary Material 11: Fig. S10E). Conversely, species-specific layer-enriched genes were identified in mouse, including *Fabp7* (L1), *Etv1* (L5), and *Tle4* (L6) (Fig. 5H). Additionally, mouse L2 and L3 were not clearly separable (Fig. 5F; Supplementary Material 11: Fig. S10D); however, we found that they were composed of two clusters, which spatially formed two distinct layers (Supplementary Material 11: Fig. S10B). Genes highly expressed in human L2 (e.g., *CALB1*, *LAMP5*) were broadly expressed across mouse L2/3 (Supplementary Material 11: Fig. S10F). Regarding cellular organization, most mouse excitatory neurons maintained distinct layer-specific enrichment patterns that aligned with GD-Ls-defined layers (Fig. 5I, J; Supplementary Material 11: Fig. S10G, H). Inhibitory neuron and non-neuronal cell distributions were relatively diffuse, and RELN neurons were sparser in mouse cortex than in primates (Supplementary Material 11: Fig. S10I).

Together, these results demonstrate that the GD-Ls method robustly delineates cortical layers in both macaque and mouse, supported by layer-enriched gene expression and cell-type organization, despite clear interspecies divergence in layer-specific molecular and cellular characteristics.

### GD-Ls exhibit high sensitivity to compromised peri-tumoral cortex

**Fig. 6:**
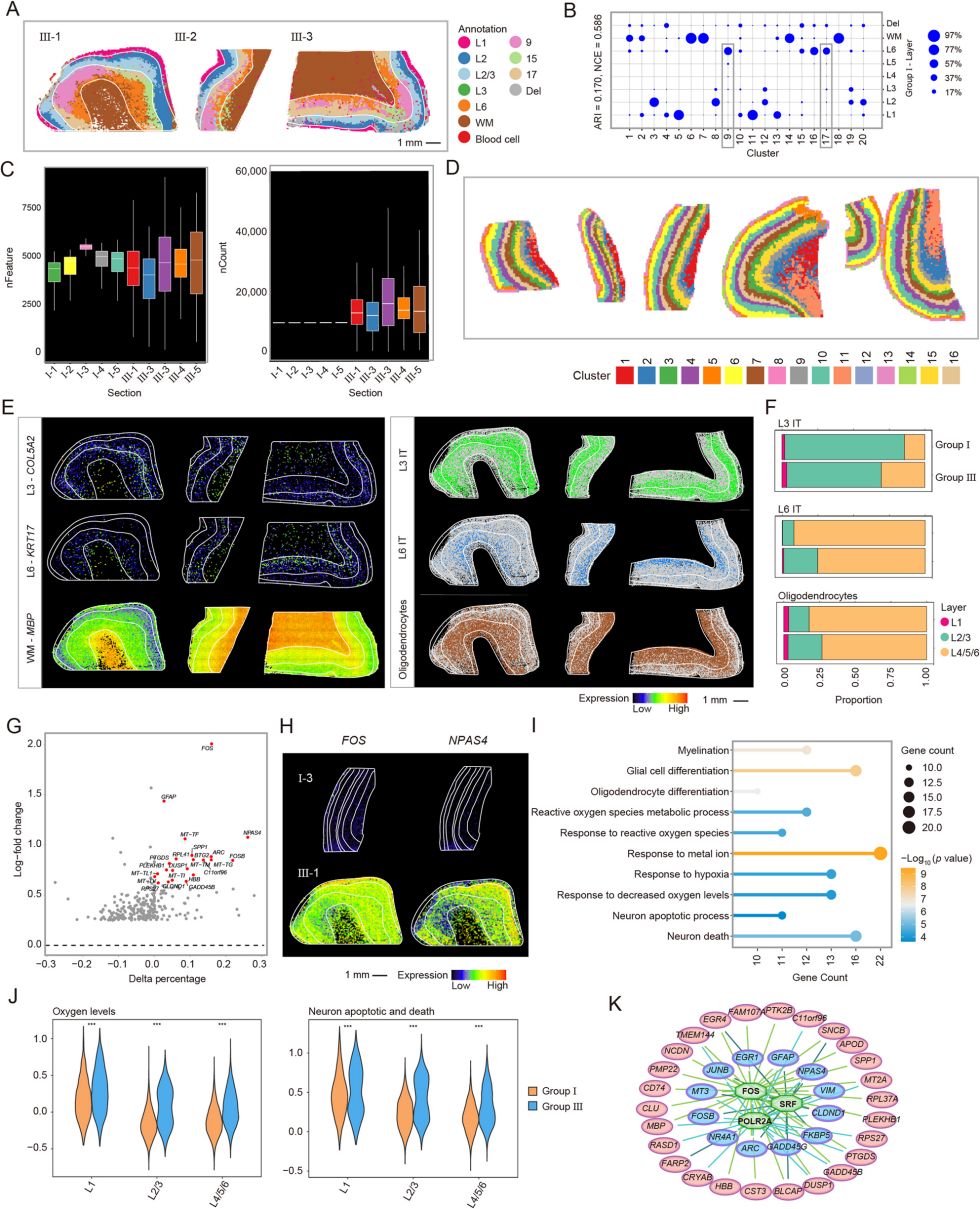
GD-Ls reveal tumor-associated disruption of cortical layers. **A** Spatial UMAP showing cortical layers identified by BayesSpace (n_clu = 20). **B** Dot plot showing the correlation between Group Ⅰ and Group Ⅲ sections. **C** Box plot showing the numbers of detected genes and total counts for Group I (after gene counts down-sampling) and Group III sections. **D** UMAP showing BayesSpace clustering of Group I after genes count down-sampling. **E** Spatial maps showing layer marker gene expression (left) and cell-type distribution (right). White lines indicate the boundaries defined by GD-Ls. **F** Bar chart showing the layer distribution percentages of each cell type in Group Ⅰ and Ⅲ sections. **G** Scatter plot depicting differentially expressed genes (DEGs) in L4/5/6 between Group Ⅰ and Group Ⅲ. Red points represent genes with log_2_FC > 0.6 and pct.1 > pct.2. **H** Spatial expression patterns of *FOS* and *NPAS4*. **I** Lollipop chart displaying GO terms enriched among DEGs in panel (**G**). **J** Violin plots showing pathway-associated gene expression across cortical layers in Group Ⅰ and Group Ⅲ. Statistical significance was assessed using unpaired *t-tests* with *p*-values adjusted using the Benjamini-Hochberg method. Significant *p*-values are indicated as follows: *p < 0.001. Data from multiple sections and technical replicates are included. **K** Network diagram showing regulatory interactions among transcription factors (TFs) associated with DEGs in panel (**G**). Three core TFs are positioned centrally; genes in the inner circle are jointly regulated by all three TFs, whereas genes in the outer circle are regulated by one or two TFs

The failure to delineate the L4/5 boundary in sections Ⅱ-3 and Ⅱ-4 from peri-tumoral tissue (Supplementary Material 9: Fig. S8E) suggested that GD-Ls capture alterations in cortical laminar gene expression and may provide a sensitive readout of peri-tumoral pathology. To test this, we applied the GD-Ls method to Group Ⅲ sections containing nubby clusters using optimized parameters (Supplementary Material 12: Fig. S11A). All 20 clusters were annotated based on marker gene expression and correlations with layer-specific signatures derived from Group Ⅰ (Fig. 6A, B; Supplementary Material 12: Fig. S11B-D). The results revealed widespread disorganization across most layers, with the exception of L1 and WM. To evaluate whether reduced gene detection influenced clustering, we performed gene count down-sampling on Group Ⅰ data to simulate the lower gene capture levels of Group Ⅲ (Fig. 6C). Subsequent clustering reproduced grouping patterns consistent with the original Group Ⅰ results (Fig. 6D; Supplementary Material 2: Fig. S2A), indicating that the impaired clustering resolution in Group Ⅲ was not attributable to limited gene detection. The expression profiles of nubby clusters 12 and 19 resembled those of L2 and L3, complicating delineation of the L2/L3 boundary (Fig. 6B; Supplementary Material 12: Fig. S11A). Meanwhile, nubby clusters 9 and 17 occupied spatial positions corresponding to L4/5 but showed the strongest transcriptional correlation with Group I L6. Cluster 15 exhibited variable spatial distributions across sections, localizing either within L4/5 regions or near the L6/WM interface, and displayed ambiguous gene expression patterns (Fig. 6A, B; Supplementary Material 12: Fig. S11A, B), thereby confounding discrimination among L4, L5, and L6. Using AD-Ls as a structural reference, we quantified the distribution of Bayes20 clusters within each layer. Compared with Group I, Group III showed significantly higher within-layer Bayes20 entropy and lower layer purity, indicating increased spatial-domain mixing and reduced laminar coherence (Supplementary Material 12: Fig. S11E). These findings provide quantitative support for the laminar disorganization observed in peri-tumoral sections. Further analysis revealed that the layer-enriched expression patterns of *COL5A2* and *KRT17* were no longer clearly maintained, with both genes showing diffuse expression across multiple cortical layers rather than distinct laminar enrichment (Fig. 6E; Supplementary Material 12: Fig. S11F). We also observed a marked increase in the proportion of L3 IT neurons within deep cortical layers, along with enhanced infiltration of L6 IT neurons and oligodendrocytes into superficial layers (L2/3) (Fig. 6E, F; Supplementary Material 12: Fig. S11G), indicating substantial laminar disruption in Group Ⅲ. Given the pronounced abnormalities in the deep cortical layers of Group Ⅲ, we performed differential gene expression analysis comparing the deep layers of Group Ⅰ and Group Ⅲ. A total of 281 genes were upregulated in Group Ⅲ deep layers (*p*_adj < 0.05 and avg_log_2_FC > 0.25), with 22 genes showing particularly strong upregulation (avg_log_2_FC > 0.6, pct.1 > pct.2) (Fig. 6G). Among these, the cellular proliferation regulators *FOS* and *FOSB*, as well as the transcription factor *NPAS4*, previously linked to neuronal plasticity and cancer cell migration [32], were more highly expressed in Group III sections than in Group I (Fig. 6H). These molecular changes are consistent with tumor-associated transcriptional alterations reported in glioblastoma-related contexts [33, 34]. Functional enrichment analysis indicated that upregulated genes in Group Ⅲ deep layers were associated with myelination, gliogenesis, hypoxia response, and neuronal apoptosis (Fig. 6I). In particular, genes involved in hypoxia and apoptosis pathways showed pan-cortical elevation in Group Ⅲ (Fig. 6J). Transcription factor regulatory analysis identified FOS, SRF, and POLR2A as core regulators of multiple upregulated differentially expressed genes. FOS has established roles in tumorigenesis [35], and SRF was highly expressed in high-grade glioma and may promote angiogenesis via vascular endothelial growth factor [36]. POLR2A, the central catalytic subunit of RNA polymerase II, was frequently mutated in meningioma and represents a risk factor for tumor recurrence (Fig. 6K) [37]. In addition, target genes of these transcription factors, such as JUNB [38, 39] and NR4A1 [40, 41], have also been implicated in tumor development and progression, functioning in proliferation, differentiation, apoptosis, and hypoxia response. Disease ontology (DO) analysis further linked highly expressed genes in Group Ⅲ deep layers with nervous system neoplasms, including neuroblastoma, high-grade glioma, and neuropathy (Supplementary Material 12: Fig. S11H). Expression analysis across cell types in both groups further revealed that multiple cell types elevated their expression of neuropathy-associated genes in Group Ⅲ (Supplementary Material 12: Fig. S11I). These findings suggest that the aberrant transcriptional profiles in Group Ⅲ sections reflect tumor-associated remodeling of the cortical landscape.

In summary, the emergence of nubby clusters in cortical sections likely reflects tumor-associated disruption of cortical lamination. This highlights the utility of the GD-Ls framework for characterizing sub histological pathological changes that may not be evident through traditional structural assessment.

## Discussion

In this study, we applied the gene-expression-defined layers (GD-Ls) method together with spatial cell-type distribution features to perform a comprehensive hierarchical analysis of human cortical layer architecture. The GD-Ls approach delineated cortical layers and sublayer-like domains across diverse human cortical sections and cross-species brain sections, and was sensitive to changes in cortical tissue state. By integrating molecular signatures with spatial cellular organization, the GD-Ls framework provides a complementary approach for cortical layer analysis. This method bridges transcriptional heterogeneity and anatomical architecture, improving the reproducibility and efficiency of laminar classification while providing a useful framework for studying neurodevelopmental disorders, neurodegenerative diseases, and brain tumor microenvironments.

While traditional anatomically guided approaches, such as Nissl-stain-directed microdissection, have established foundational classifications of cortical layers, they may be limited by subjective boundary assignment and technical variability. By contrast, data-driven approaches based on layer-enriched gene expression provide an alternative framework for laminar parcellation and can reveal molecularly distinct spatial domains. However, the alignment between these data-driven laminar boundaries and anatomically defined layers remains insufficiently validated, particularly in the human cortex, where layer thickness varies across different lobes [42, 43], warranting further investigation. Our analysis reveals that GD-Ls delineate cortical layers, including both linear and curved laminae, with improved efficiency and boundary definition relative to traditional anatomy-defined layer parcellation (AD-Ls). This framework also facilitated the simultaneous analysis of multiple tissue sections, improving the efficiency and reproducibility of layer identification. Validation using independent healthy postmortem datasets confirmed that GD-Ls stably recover major laminar organization across tissue sources and platforms, indicating that our framework captures intrinsic cortical architecture. Notably, validation in 10x Visium data suggests that GD-Ls are not restricted to a single platform. However, their performance likely depends on spatial resolution and transcriptome coverage. While sequencing-based platforms such as Stereo-seq and Visium are well-suited for laminar delineation, imaging-based platforms with limited gene panels (e.g., MERFISH and Xenium) may have reduced capacity to resolve fine laminar boundaries. Despite interspecies differences in layer-specific gene expression, GD-Ls enabled rapid cross-species layer classification using species-specific layer-enriched gene sets. Furthermore, GD-Ls appeared more effective than AD-Ls in detecting pathological laminar disruptions, thereby improving the localization of lesioned regions in diseased cortical tissues.

GD-Ls resolved the canonical layers (L1-L6 and WM) while also revealing finer sublayer-like heterogeneity. L6b, recognized as the deepest neocortical layer, resides at the border between GM and WM [44]. Using GD-Ls, we delineated the L6b sublayer and additionally observed a WM-associated compartment (WM_a) located between L6b and deeper WM, showing hybrid molecular features of both GM and WM. Given its mixed molecular profile and its position at the GM-WM interface, WM_a is more conservatively interpreted as a SWM-like transitional zone, or a gray-white matter transitional compartment, rather than a fully independent canonical layer. This interpretation is consistent with the possibility of a continuous interface between L6b and adjacent superficial white matter [22–24]. More broadly, although GD-Ls revealed several sublayer-like domains, their precise biological roles and functional significance remain unclear at present and should be interpreted cautiously. Future studies with higher spatial resolution and orthogonal validation will be needed to resolve their cellular composition and clarify their anatomical and biological significance.

Brain tumors can disrupt cortical organization beyond overt lesional regions [45–54]. In this study, GD-Ls detected laminar disorganization and molecular alterations in cortical regions that appeared histologically unremarkable, suggesting that this approach may be sensitive to pathological changes not readily captured by conventional histology. These findings indicate that molecular and microstructural abnormalities are present in cortical regions lacking overt histological alterations, underscoring the potential of GD-Ls to identify subtle disease-associated changes in cortical architecture. However, the temporal relationship and causal significance of these changes remain to be determined. Future studies, particularly those incorporating longitudinal designs, will be needed to assess whether distinct tumor types, neurodegenerative disorders, or inflammatory diseases are associated with different patterns of cortical disruption. Integrating multi-omics approaches with GD-Ls-based spatial profiling may further elucidate the mechanistic links between molecular alterations and clinical outcomes, including disease progression, therapeutic responses, and functional impairments. Such integrated analyses may help clarify disease-associated cortical disruptions and their pathological relevance. For potential clinical applications, such as surgical planning, further validation in larger, well-annotated cohorts with spatially resolved distance-to-lesion information will be required. In addition, quantitative metrics of laminar disorganization, such as boundary sharpness and marker coherence, will be important for future clinical translation.

Our analysis of both linear and curved cortical regions reveals the intricate laminar complexity of the human cerebral cortex. The human brain features an elaborate system of gyri and sulci [55, 56], but the molecular heterogeneity across these structures and cortical lobes, and its relationship to biological function, remain poorly understood. Although spatially resolved transcriptomic datasets have been generated for entire brain sections in non-human primates and rodents [6, 7, 57–59], comprehensive profiling of the entire human brain has not yet been achieved due to its unparalleled structural complexity. In the present study, laminar delineation was primarily based on conserved layer-associated gene expression patterns, and our sample size was not sufficient to systematically resolve inter-lobar differences. Therefore, potential lobe-specific variation in cortical laminar organization remains an important question for future investigation. Acquiring larger-scale datasets from intact human cortical specimens across multiple lobes would provide a critical foundation for determining which aspects of laminar architecture are broadly conserved and which show region- or lobe-specific specialization. Future research should extend this framework to investigate how genetic, developmental, or disease-related factors regulate the layered architecture of highly convoluted cortical regions across distinct cortical areas. Integrating multi-omics approaches will be essential for deciphering the mechanisms that govern the brain’s multifaceted functional organization. Combining spatial transcriptomics with complementary datasets, such as connectomics, epigenomics, and functional activity data, could reveal how molecular, structural, and functional interactions collectively orchestrate cognition and behavior. Such integrative analyses may identify cross-scale hubs where genetic variants, developmental processes, or pathological perturbations converge to shape cortical layer specialization. This holistic approach promises to bridge molecular signatures with functional neuroanatomy, advancing our understanding of the human brain’s unique complexity.

## Conclusions

In summary, the GD-Ls framework establishes a robust, gene-expression-based system for cortical layer identification that transcends the limitations of anatomy-driven approaches. Its consistency across diverse cortical regions and multiple species, including human, macaque, and mouse, underscores its broad applicability and positions it as a scalable tool for large-scale neuroanatomical studies. Meanwhile, GD-Ls reveal molecular-morphological dissociation in cortical tissues that appear histologically unremarkable, enabling sensitive detection of pathological alterations in disorders such as brain tumors. By integrating molecular resolution with anatomical context, GD-Ls not only refine our understanding of cortical organization but also open new avenues for translational research in clinical neuropathology.

## Supplementary Information

Supplementary Material 1. Supplementary tables. (Table S1-S4)

Supplementary Material 2. Supplementary figure. (Fig. S1)

Supplementary Material 3. Supplementary figure. (Fig. S2)

Supplementary Material 4. Supplementary figure. (Fig. S3)

Supplementary Material 5. Supplementary figure. (Fig. S4)

Supplementary Material 6. Supplementary figure. (Fig. S5)

Supplementary Material 7. Supplementary figure. (Fig. S6)

Supplementary Material 8. Supplementary figure. (Fig. S7)

Supplementary Material 9. Supplementary figure. (Fig. S8)

Supplementary Material 10. Supplementary figure. (Fig. S9)

Supplementary Material 11. Supplementary figure. (Fig. S10)

Supplementary Material 12. Supplementary figure. (Fig. S11)

## Data Availability

The spatial transcriptomics sequencing data generated from the 15 human cortex sections analyzed in this study have been deposited in the China National GeneBank Sequence Archive (CNSA) under accession code CNP0009546 and are available at https://db.cngb.org/data_resources/project/CNP0009546 [10, 60]. Previously published datasets used in this study are available from the Gene Expression Omnibus (GEO) under accession codes GSE269906 (https://www.ncbi.nlm.nih.gov/geo/query/acc.cgi?acc=GSE269906) [26] and GSE307403 (https://www.ncbi.nlm.nih.gov/geo/query/acc.cgi?acc=GSE307403) [22], and from STOmicsDB under accession code STDS0000242 (https://db.cngb.org/stomics/datasets/STDS0000242/summary). The source code implementing the GD-Ls pipeline, including scripts for data preprocessing, parameter selection, clustering, GD-Ls annotation, and validation analyses, is publicly available on GitHub at https://github.com/YanrongWei/GD-Ls [61, 62]. The released version of the source code has also been archived in Zenodo and can be cited using the DOI: 10.5281/zenodo.20199715.
