# Supplementary figures and images for: Gene expression profiling enables refined parcellation of cortical layers in the heterogeneous human cerebral cortex
Source: Genome Med. 2026 Jul 8;18:104. doi: 10.1186/s13073-026-01704-z (PMC13360882; doi:10.1186/s13073-026-01704-z)

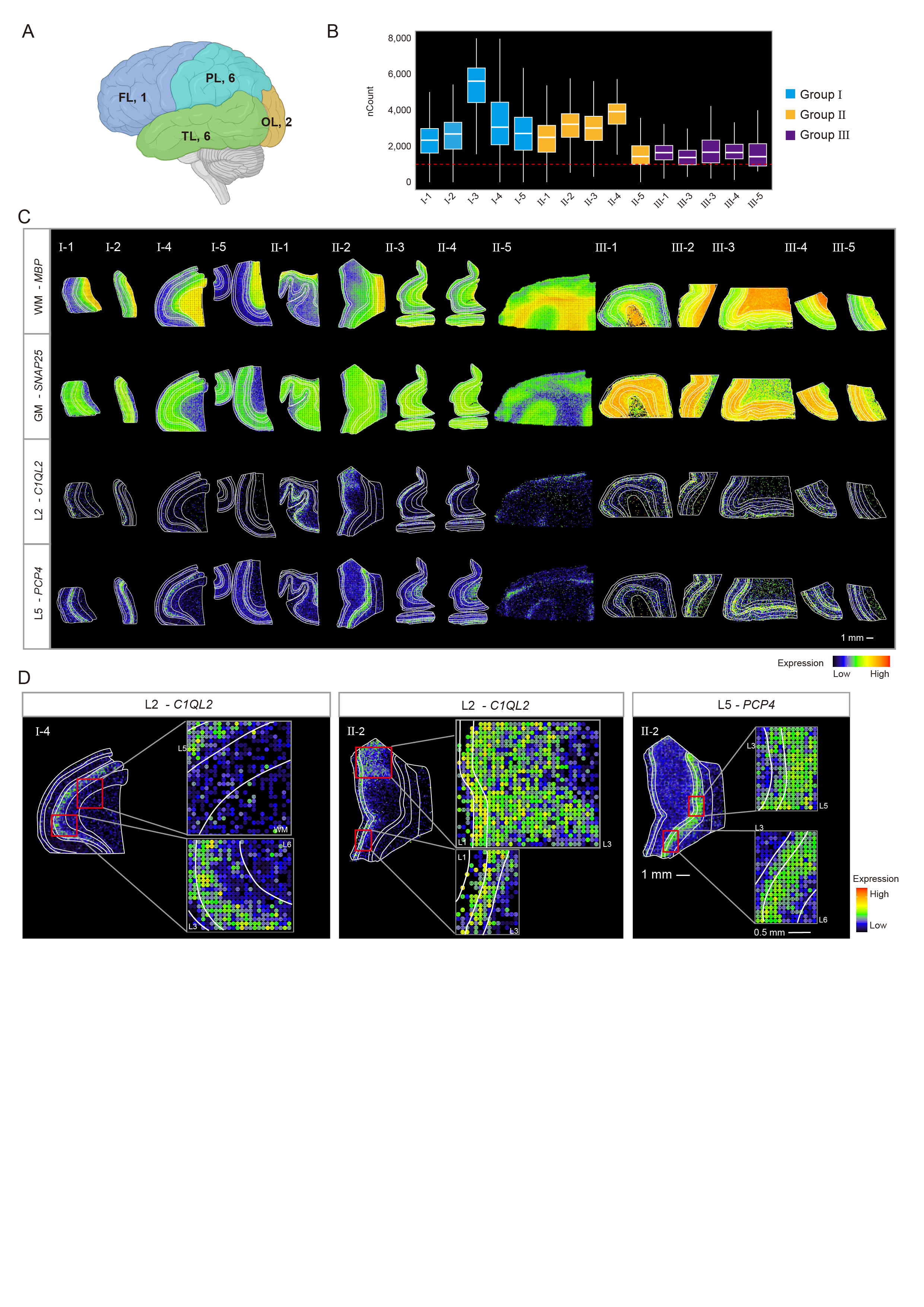

Supplement: Supplementary file 2 — Supplementary Material 2. Supplementary figure. (Fig. S1) [file 13073_2026_1704_MOESM2_ESM.jpg]

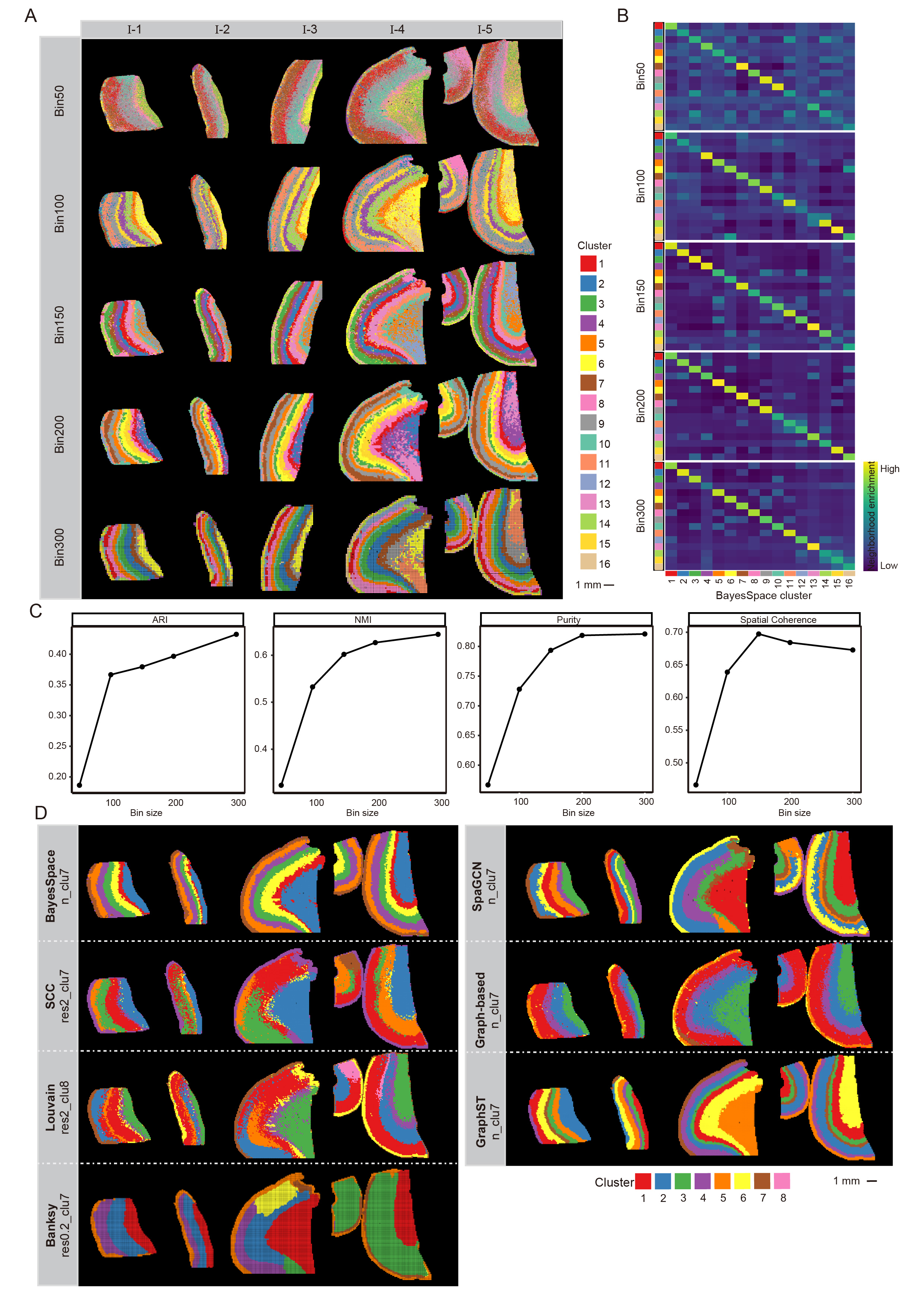

Supplement: Supplementary file 3 — Supplementary Material 3. Supplementary figure. (Fig. S2) [file 13073_2026_1704_MOESM3_ESM.jpg]

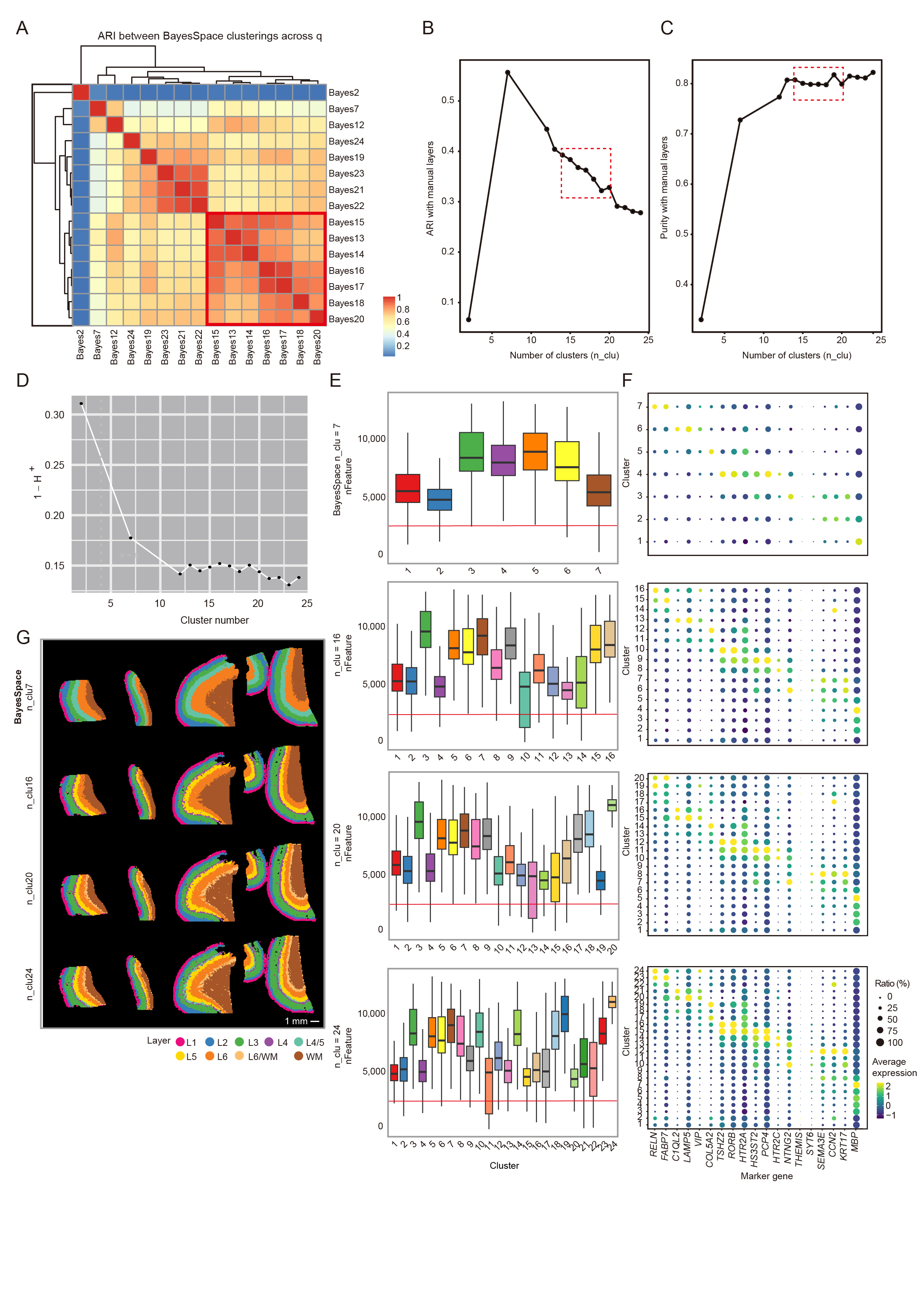

Supplement: Supplementary file 4 — Supplementary Material 4. Supplementary figure. (Fig. S3) [file 13073_2026_1704_MOESM4_ESM.jpg]

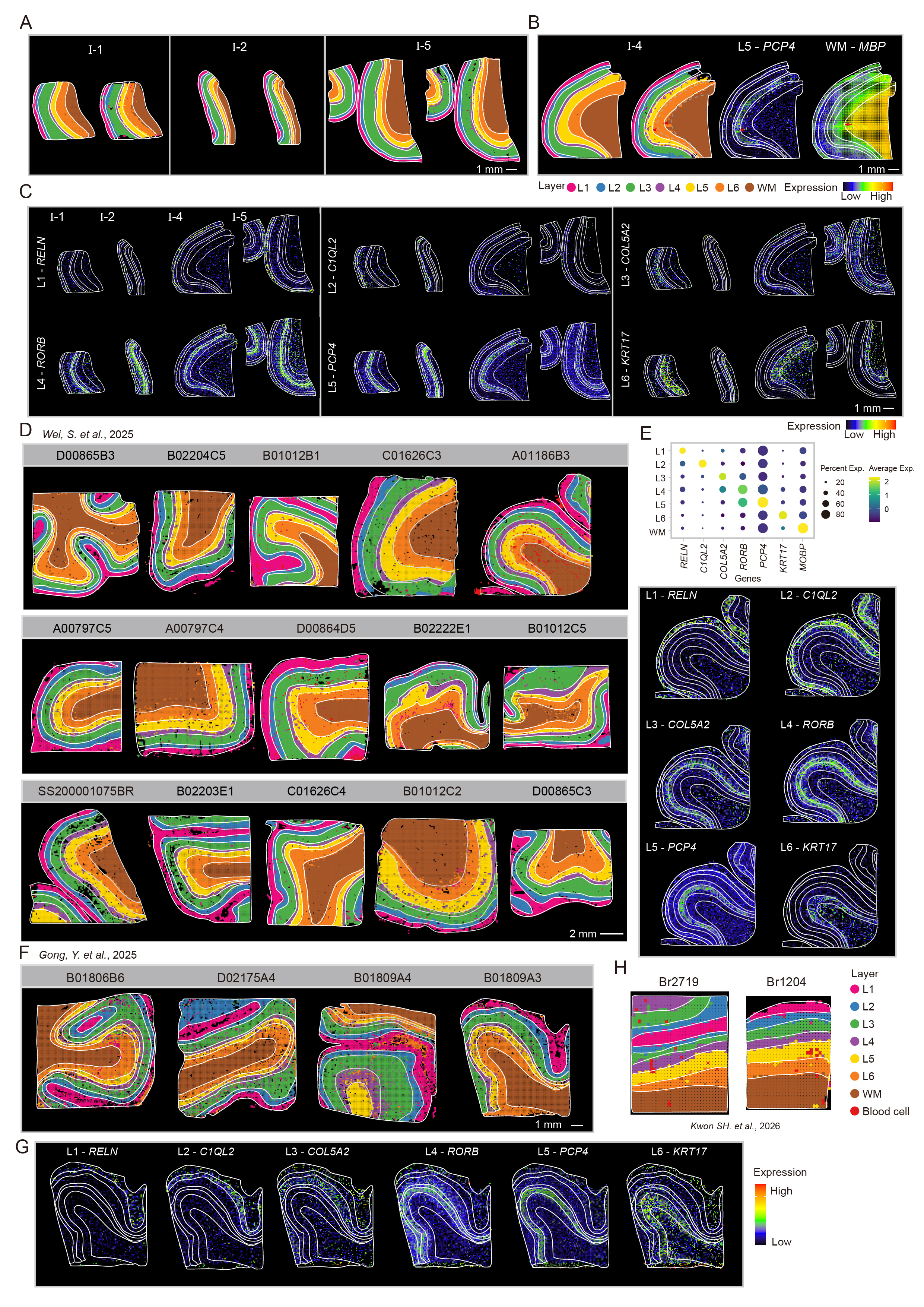

Supplement: Supplementary file 5 — Supplementary Material 5. Supplementary figure. (Fig. S4) [file 13073_2026_1704_MOESM5_ESM.jpg]

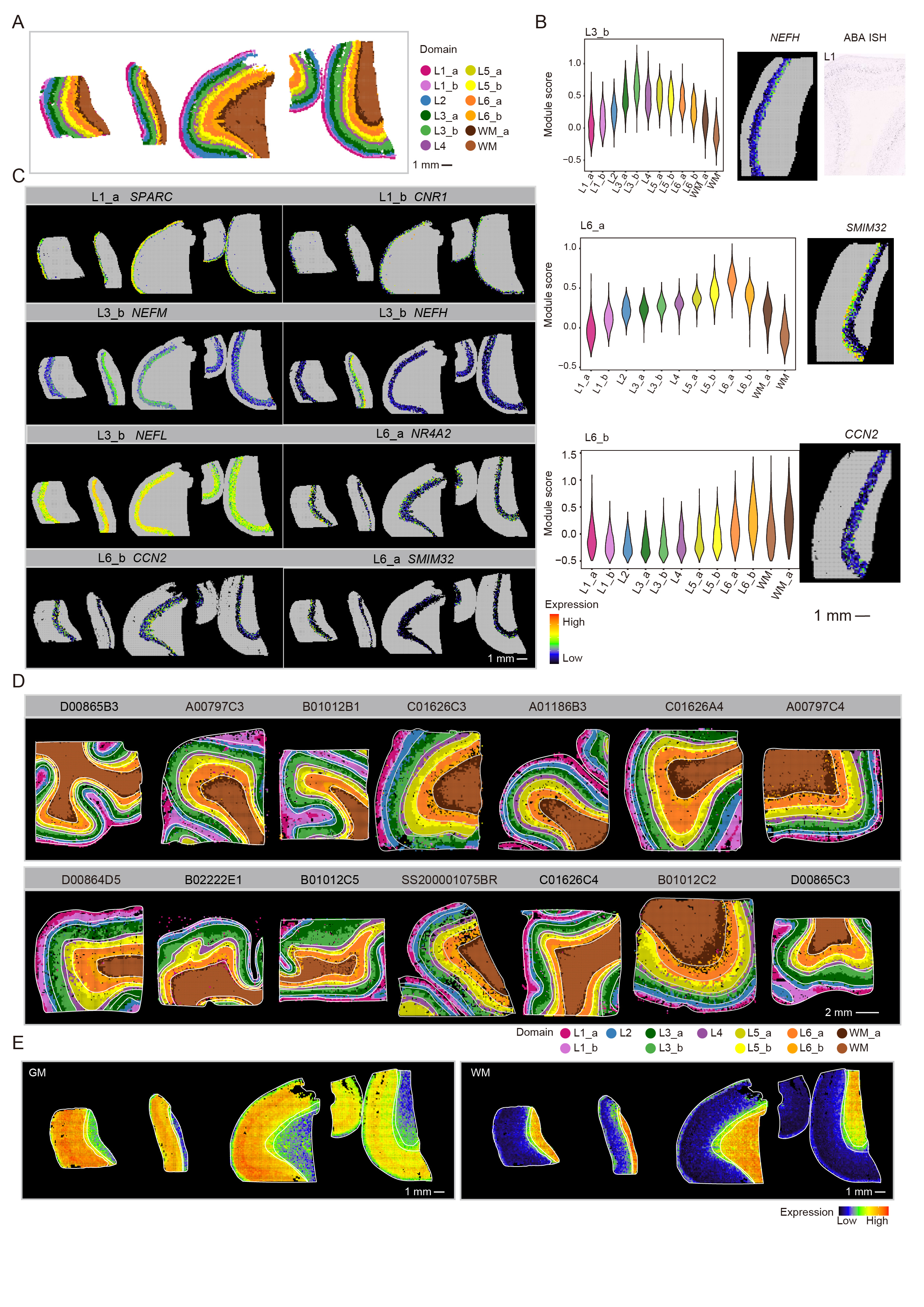

Supplement: Supplementary file 6 — Supplementary Material 6. Supplementary figure. (Fig. S5) [file 13073_2026_1704_MOESM6_ESM.jpg]

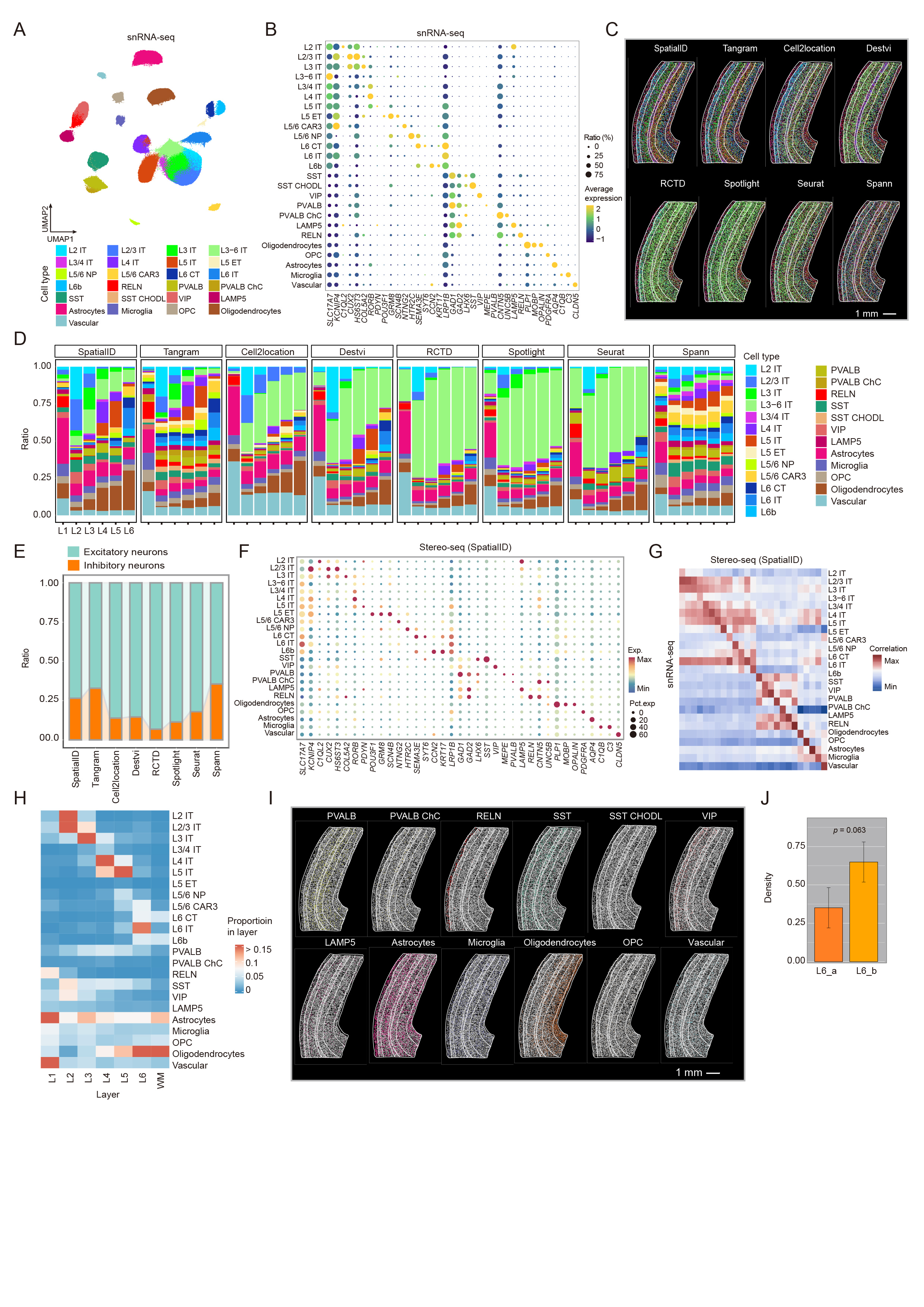

Supplement: Supplementary file 7 — Supplementary Material 7. Supplementary figure. (Fig. S6) [file 13073_2026_1704_MOESM7_ESM.jpg]

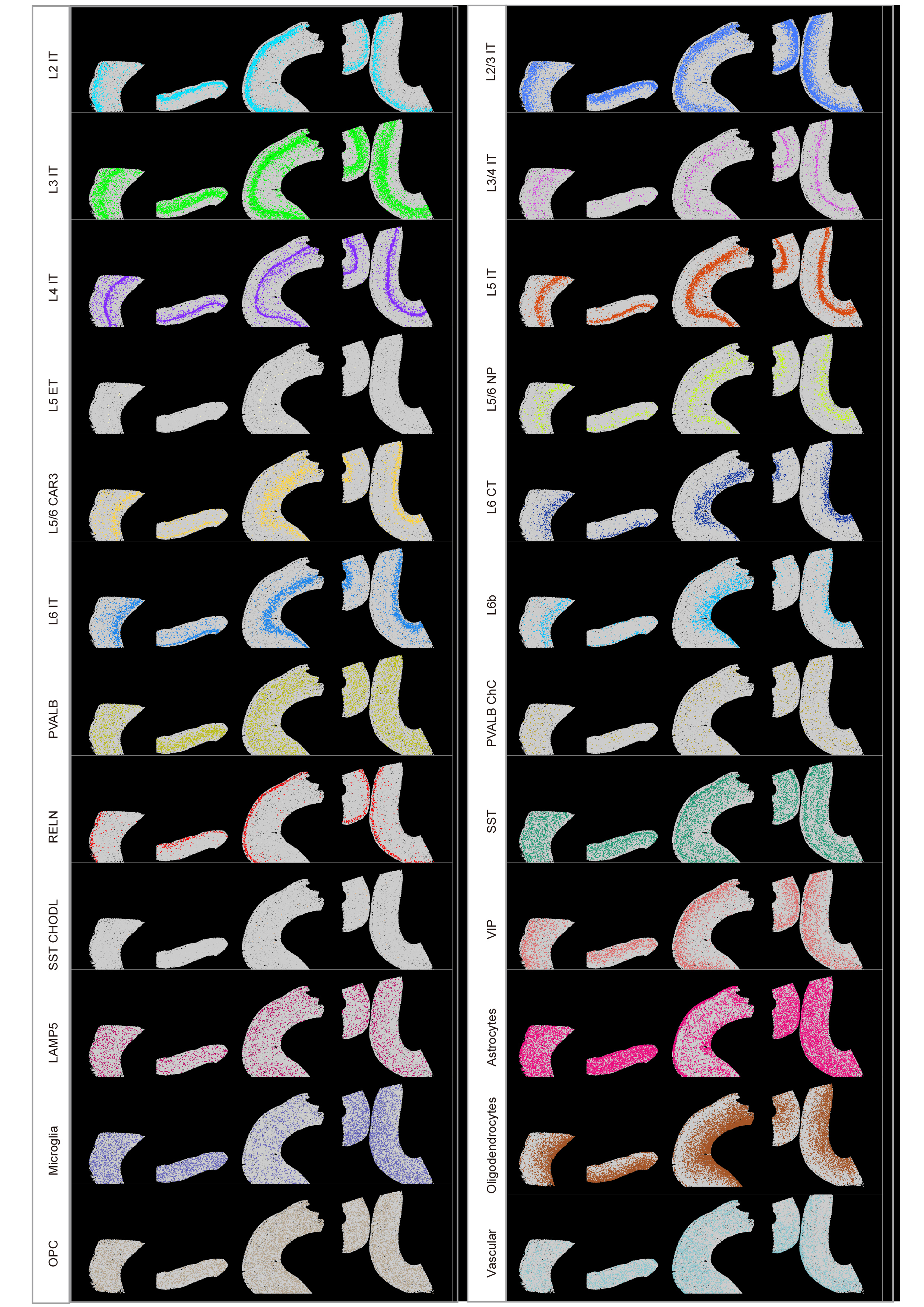

Supplement: Supplementary file 8 — Supplementary Material 8. Supplementary figure. (Fig. S7) [file 13073_2026_1704_MOESM8_ESM.jpg]

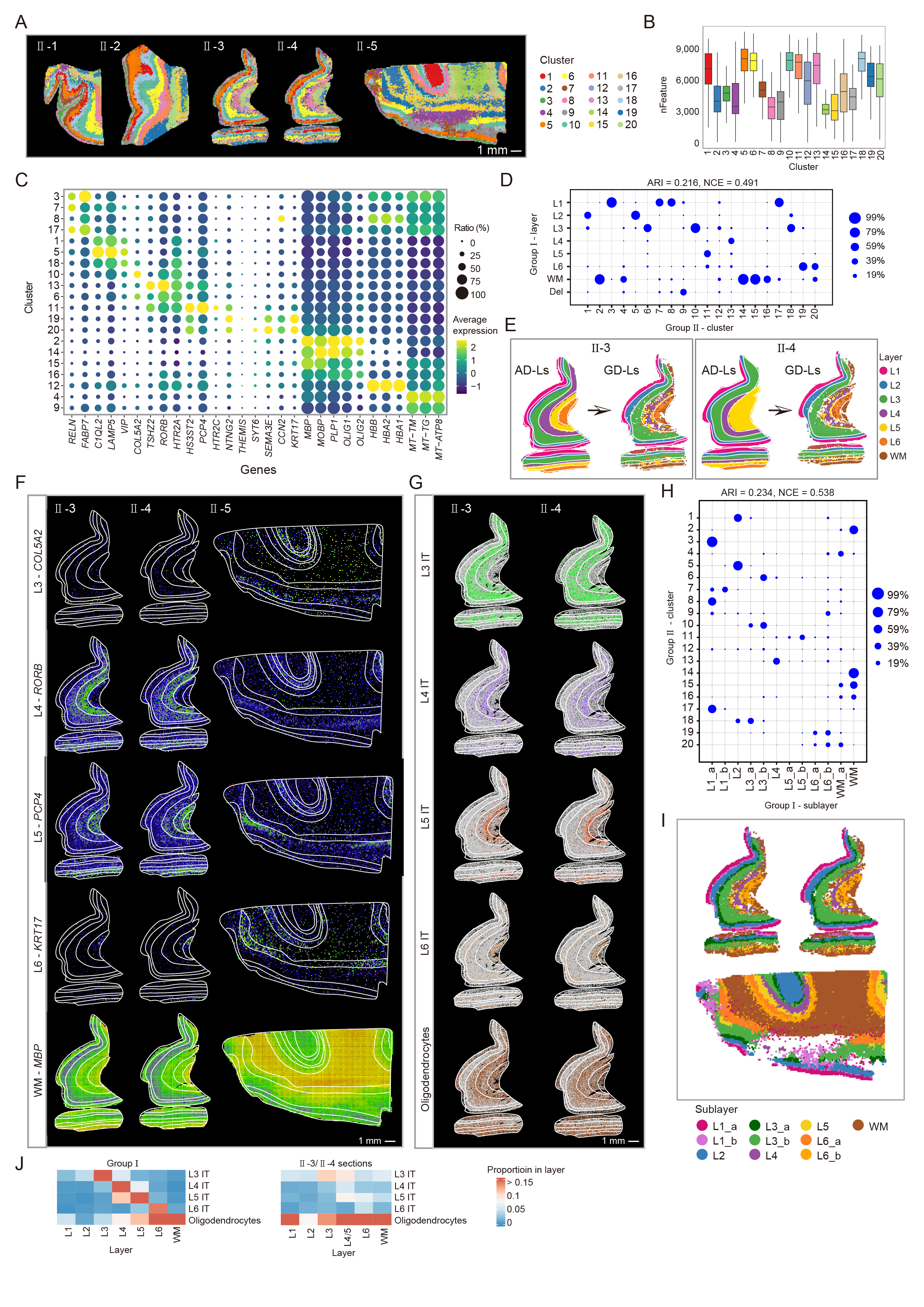

Supplement: Supplementary file 9 — Supplementary Material 9. Supplementary figure. (Fig. S8) [file 13073_2026_1704_MOESM9_ESM.jpg]

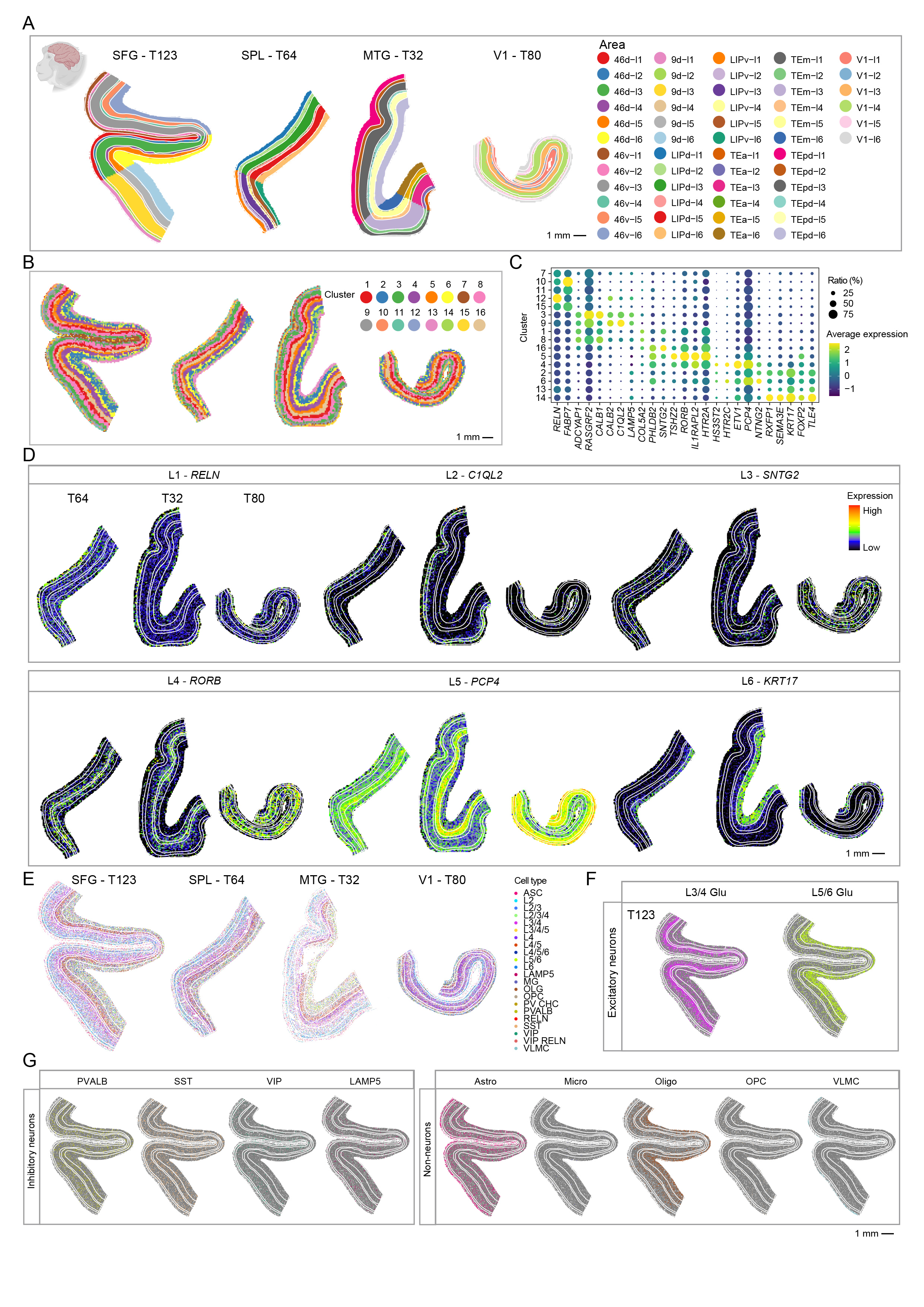

Supplement: Supplementary file 10 — Supplementary Material 10. Supplementary figure. (Fig. S9) [file 13073_2026_1704_MOESM10_ESM.jpg]

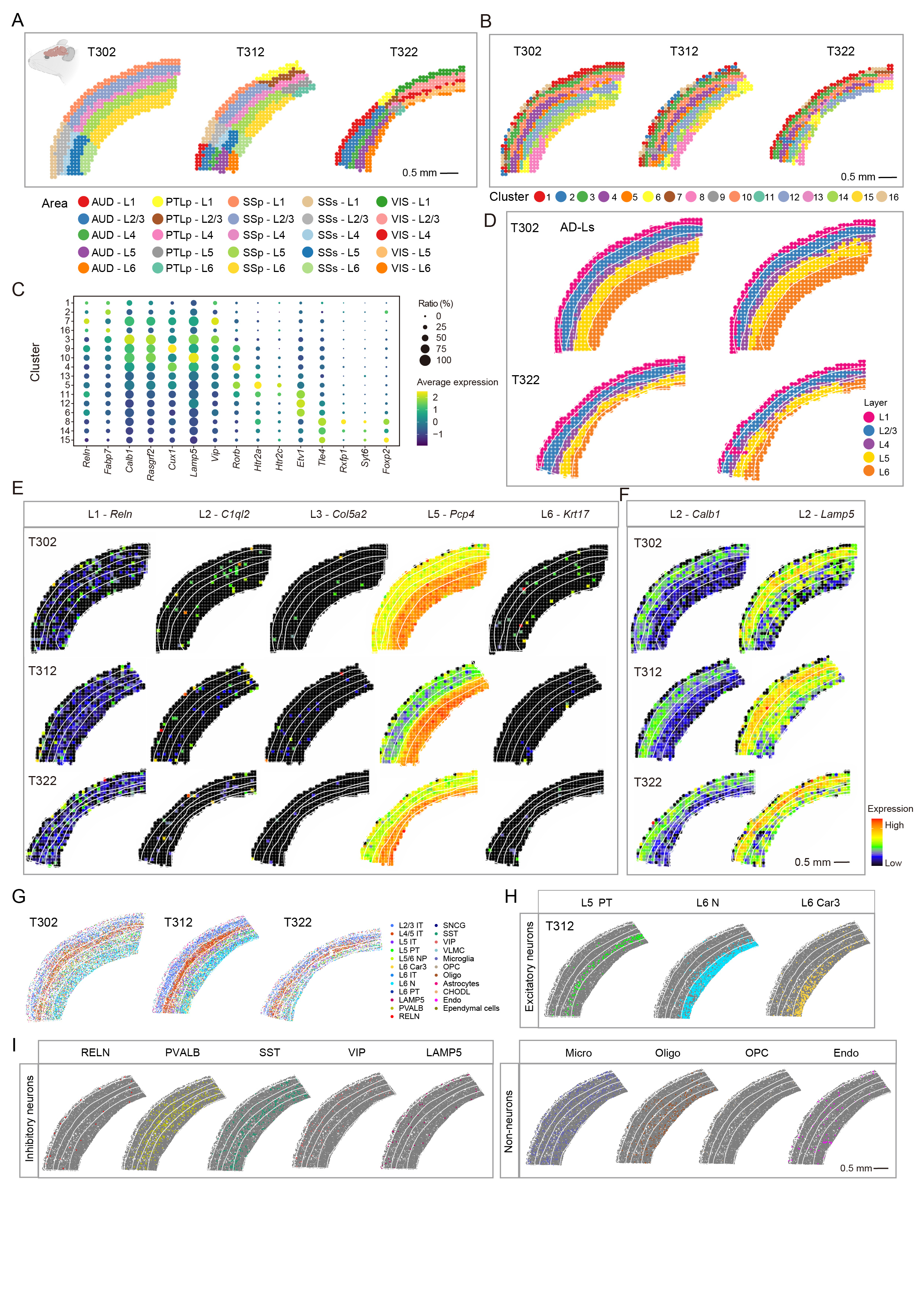

Supplement: Supplementary file 11 — Supplementary Material 11. Supplementary figure. (Fig. S10) [file 13073_2026_1704_MOESM11_ESM.jpg]

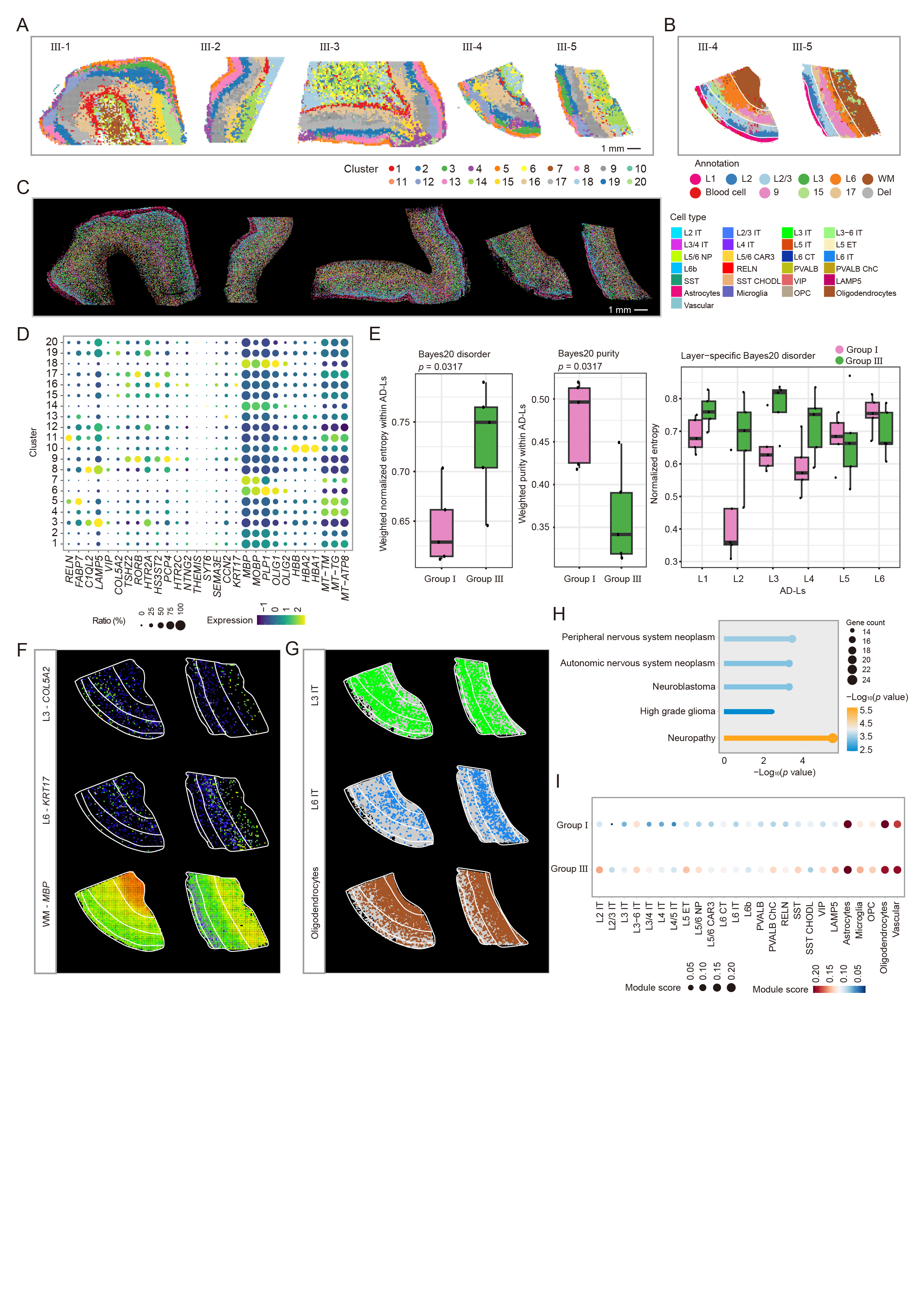

Supplement: Supplementary file 12 — Supplementary Material 12. Supplementary figure. (Fig. S11) [file 13073_2026_1704_MOESM12_ESM.jpg]
